# Longitudinal Tracking of Astrocyte Reactivity During the Development of Chronic Orofacial Neuropathic Pain Using [ ^18^F]‐SMBT‐1 Positron‐Emission Tomography

**DOI:** 10.1002/glia.70182

**Published:** 2026-06-18

**Authors:** Lewis S. Crawford, James W. M. Kang, OIivia I. Davanzo, Hyunsol Lim, Sabrina Salberg, Marissa Srgo, Crystal Li, Gaelle M. Emvalomenos, Bianca Jupp, Matthew Long, Wayne Noonan, Mohammad B. Haskali, Richelle Mychasiuk, Kevin A. Keay, Luke A. Henderson

**Affiliations:** ^1^ School of Medical Sciences [Neuroscience] The University of Sydney Sydney New South Wales Australia; ^2^ Brain & Mind Centre The University of Sydney Camperdown New South Wales Australia; ^3^ Department of Neuroscience, Central Clinical School Monash University Melbourne Victoria Australia; ^4^ The Radiopharmaceutical Research Laboratory The Peter MacCallum Cancer Centre Melbourne Victoria Australia

**Keywords:** chronic constriction injury, chronic pain, GFAP, glia, immunohistochemistry, monoamine oxidase B, nucleus accumbens, rat, spinal trigeminal nucleus, trigeminal nerve

## Abstract

Chronic neuropathic pain represents a significant global health burden and is hypothesized to be maintained by an altered astrocyte‐neuron interplay. Evidence of astrocyte contributions to neuropathic pain are derived from both post‐mortem immunohistochemical analyses and in vivo pharmacological studies. These data cannot provide insights into dynamic changes in astrocytes within an individual as pain develops. Here, we present the first use of [^18^F]‐SMBT‐1, a radioisotope that binds monoamine oxidase‐B (MAO‐B) in reactive astrocytes, in a longitudinal (35 days) study of the development of orofacial neuropathic pain. We used the chronic constriction injury of the infraorbital nerve (ION‐CCI) to produce orofacial neuropathic pain in adult male Sprague–Dawley rats, which we compared to both sham‐injury and anesthesia controls. Between 14 and 28 days following ION‐CCI, increases in SMBT‐1 binding occurred in: the trigeminal nerve, ganglion and the spinal trigeminal nucleus; as well as the nucleus accumbens and dorsal striatum. Increased MAO‐B expression in these regions was confirmed using immunohistochemical analysis. MAO‐B expression was also confirmed in the ION, the trigeminal ganglion, and trigeminal root entry zone. These data provide evidence of regional and temporally specific increases in astrocyte reactivity, which may be critical for establishing chronic orofacial neuropathic pain. Longitudinal mapping of changes in astrocyte reactivity during the development of chronic orofacial neuropathic pain will provide an opportunity to test the potential of astrocyte specific compounds delivered at specific time points following nerve injury, to determine the key brain regions that could be targeted to prevent and/or reverse the development of chronic neuropathic pain.

## Graphical abstract

Using [18F]‐SMBT‐1 PET, we longitudinally tracked MAO‐B–expressing reactive astrocytes during orofacial neuropathic pain development in rats, revealing region‐ and time‐specific astrocyte activation during pain chronification.

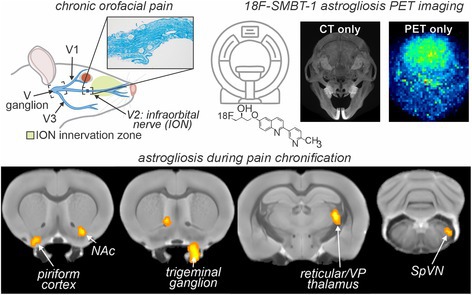

## 1 Introduction

Chronic pain imposes enormous economic and social burdens, ranked globally as the leading cause of years lived with disability (James et al. 2018), with treatment‐seeking for each individual sufferer reaching costs in excess of $US15,000 per year (Groenewald et al. 2014). Compounding this, current management strategies for chronic pain remain inadequate, with 50%–60% of individuals not receiving significant relief from first‐line pharmacological treatments (Backonja and Woolf 2010). Critically, once pain has become established, it is extremely difficult to treat, particularly chronic pain that results from nervous system damage, that is, neuropathic pain. While chronic neuropathic pain in the orofacial region often results from accidental trauma, it can also occur as a consequence of routine dental surgery such as tooth extractions, which are estimated to result in the development of chronic orofacial neuropathic pain at a rate of 3%–15% (Eliav and Benoliel 2014). This form of chronic pain is particularly debilitating and the resulting functional impairment of the face and mouth impacts both verbal and non‐verbal communication leading to psychosocial distress, and also impaired taste, olfaction, chewing, and swallowing, leading to altered appetite and ingestive functions (Sessle 2006). Our lack of understanding of the complex neurobiological changes that occur during the transition to chronic neuropathic pain underlies the significant failures in developing effective long‐term therapies for this pain condition. Preclinical models of neuropathic pain play an important role in understanding this complexity, and are critical for defining at the cellular level, the neural and non‐neural changes triggered by nerve injuries. Transection, compression, or constriction of branches of the trigeminal nerve provide a clinically relevant model of human trigeminal neuropathic pain (Iwata and Sessle 2019; Sadighparvar et al. 2023), damaged axons and Schwann cells release inflammatory mediators, and immune cells migrate to the injury site. These processes result in increased spontaneous and evoked firing of the trigeminal nerve (Kanamori et al. 2007; Peng et al. 2016). In addition, in the trigeminal ganglion immune cells accumulate around the cell bodies of the damaged axons (Donegan et al. 2013; Iwai et al. 2021). Trigeminal nerve injury triggers changes in both damaged and undamaged nerve fibers and in the spinal trigeminal nucleus (SpVN) where these fibers terminate (Ma et al. 2012). Further, altered activity in the SpVN drives changes in supra‐medullary pain recipient regions (Vos and Strassman 1995; Dubner and Ren 2004; Saito et al. 2017; Okada et al. 2019).

Cross‐sectional immunohistochemical studies in both mice and rats have shown that trigeminal nerve injuries trigger activation of microglia in the SpVN (Piao et al. 2006; Okada‐Ogawa et al. 2009; Asano et al. 2020). While microglia release cytokines that increase neuronal excitation (Coull et al. 2005; Kawasaki et al. 2008), inhibition of microglia does not consistently attenuate ongoing pain in preclinical models (Piao et al. 2006; Zama et al. 2019), therefore chronic neuropathic pain is unlikely to be microglia‐dependent. We have recently shown using positron emission tomography (PET) that chronic constriction injury of the infraorbital nerve (ION‐CCI) evokes an immediate increase in TSPO binding in the SpVN, reflecting microglial activation, but these increases in TSPO binding diminished as chronic pain developed (Emvalomenos et al. 2025). Microglial activation drives functional changes in co‐located astrocytes, and cross‐sectional immunohistochemical studies have reported that trigeminal nerve injuries increase astrocyte reactivity in the SpVN (Okada‐Ogawa et al. 2009, Asano et al. 2020). Evidence of sustained astrocyte reactivity in people with neuropathic pain has been seen in post‐mortem studies of people with HIV‐related neuropathic pain who showed significant numbers of reactive astrocytes, but not microglia, in the spinal cord dorsal horn (Shi et al. 2012). Increased astrocyte reactivity can result in increased neural excitability in pain processing circuits resulting in chronic neuropathic pain. Attenuating astrocyte reactivity in the SpVN that results in a reduction of glutamine production, can strongly decrease trigeminal nerve injury‐evoked pain (Okada‐Ogawa et al. 2009; Tsuboi et al. 2011). There is no evidence for sustained neural activity in the central pain pathways of people with chronic neuropathic pain (Iadarola et al. 1995; Youssef et al. 2014), however in people with trigeminal neuropathic pain there is evidence of increases oscillatory activity in the SpVN (Alshelh et al. 2016). The frequencies of these signals are similar to calcium waves in reactive astrocytes (Scemes and Giaume 2006), and it has been hypothesized that nerve injury, alters astrocyte reactivity resulting in altered glio‐transmission and thus astrocyte‐neural coupling (Henderson and Di Pietro 2016). This effect is also observed in SpVN recipient parts of the thalamus, where these slow neural rhythms may underpin thalamocortical dysrhythmia via cross‐frequency coupling which, via an unknown mechanism, results in the constant perception of pain. Current understanding of the contribution of astrocyte reactivity to the development of neuropathic pain is derived from cross‐sectional studies and a majority are static snapshots from post‐mortem histological/immunohistochemical analyses.

**FIGURE 1:**
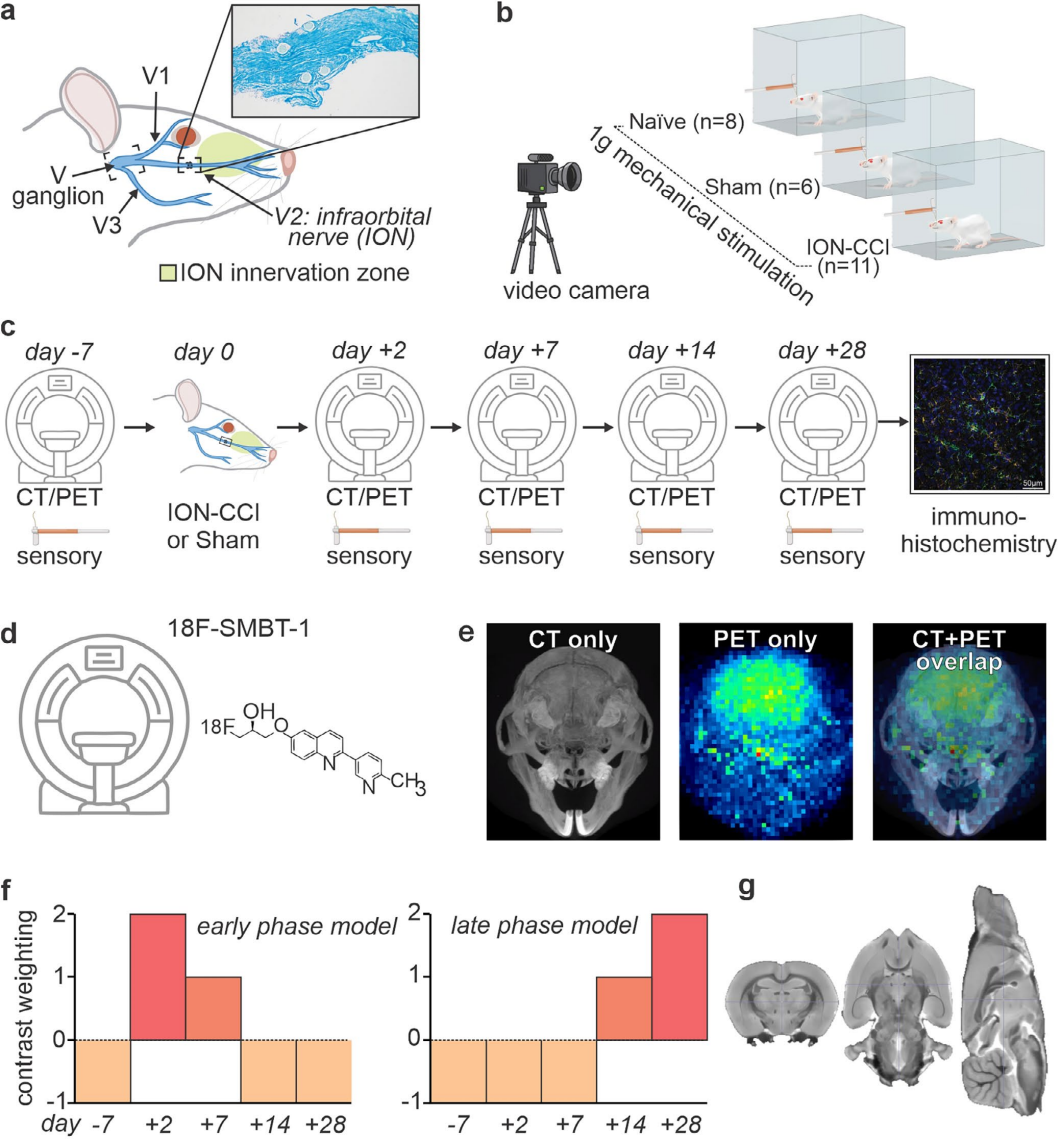
Experimental methodology, imaging acquisition and analysis. (a) ION‐CCI rats, in which two ligatures are tied approximately 2 mm apart around the nerve (~40 min). Sham rats have identical surgery, but no ligatures applied (~40 min), and Naïve rats were anesthetized (~40 min). (b) Mechanical sensory testing consisted of application of a 1‐g von‐Frey filament to the skin innervated by the ION. Behavioral responses were video‐recorded and scored as described in Kang et al. (2024). (c) Baseline CT/PET acquisition occurred 7 days before surgery (day −7); subsequent CT/PET acquisitions were performed on days 2, 7, 14, and 28 post‐surgery. On day 30, rats were deeply anesthetized and perfused with fixative for tissue processing. (d) Under anesthesia, dynamic PET images were acquired over 60 min using [^18^F]‐SMBT‐1. (e) CT was visualized in the PMOD software with PET data checked for registration and field of view cropped to match the CT. The final three frames (30 min) of dynamic PET were summed and converted to standardized uptake value ratio contrast images. CT data were next transformed to the SIGMA atlas template (g), with parameters applied to the PET data. Group‐level analyses were conducted using statistical parametric mapping within Matlab on SUVr images of the ION‐CCI cohort. (f) Full factorial analysis with a single factor of five layers representing each experimental time point was constructed, with contrasts generated which either highlighted significant changes at later experimental time points “*late* phase model” (left), or immediately after injury “*early* phase model” (right). In both models, clusters demonstrating significant change in [^18^F]‐SMBT‐1 binding were saved, and values extracted from both Sham and Naïve cohorts to determine the specificity of these changes.

A critical need for our understanding of the transition from acute to chronic neuropathic pain are studies of the dynamic changes in astrocyte‐neural activity in an individual, as pain develops, or in response to interventions that attenuate astrocyte activity. In this study we used PET imaging with the recently developed radiotracer, selective to monoamine oxidase‐B (MAO‐B): (*S*)‐(2‐methylpyrid‐5‐yl)‐6‐[(3‐^18^F‐fluoro‐2‐hydroxy)propoxy]quinoline ([^18^F]‐SMBT‐1), in a longitudinal design to visualize astrocyte reactivity in vivo, during the development of trigeminal neuropathic pain in the rat. Currently, [^18^F]‐SMBT‐1 is the most suitable radiotracer for the longitudinal study of astrocytes in vivo due to its favorable binding characteristics and validated specificity (Emvalomenos et al. 2024). Further, its verified binding accuracy to MAO‐B in humans makes its use attractive for cross‐species translational studies. Using the ION‐CCI model (Vos and Strassman 1995) (Figure 1A), we describe temporal changes in [^18^F]‐SMBT‐1 binding in male rats, prior to, and up to 28 days after ION‐CCI compared to sham‐injured and naïve anesthetized counterparts (Figure 1B–E). Sites of significantly altered [^18^F]‐SMBT‐1 binding ratio (i.e., SUVr) were evaluated post‐mortem using immunohistochemistry to identify the tissue distribution of both MAO‐B and glial fibrillary acidic protein (GFAP) in the infraorbital nerve, trigeminal ganglion, and throughout the brain. We hypothesized a nerve injury to evoke increases in [^18^F]‐SMBT‐1 binding and MAO‐B expression in infraorbital nerve recipient regions of the brainstem, specifically in the SpVN, and in SpVN recipient supra‐medullary brain regions. Further, we hypothesized a temporal difference in ^18^F‐SMBT‐1 binding and MAO‐B expression, with an “early” brainstem and “later” forebrain pattern of expression.

## 2 Materials and Methods

All experimental procedures were carried out with the approval of Alfred Medical Research and Education Precinct Animal Ethics Committee at the Precinct Animal Centre (PAC; E/8283/2022/M) and in accordance with the guidelines of the Code for the Care and Use of Animals in Research Australia, and IASP Guidelines for the Use of Animals in Research (Zimmermann 1983).

### 2.1 Experimental Design

Adult male Sprague–Dawley rats (*n* = 25) at 6 weeks of age were sourced from the Monash Animal Research Platform and kept for the duration of the experiment on a 12:12 h light/dark cycle in a temperature‐controlled environment. Rats were housed 2–3 per cage with *ad libitum* access to standard laboratory chow and water. Rats were handled and weighed regularly (4–7 times/week). Following acclimatization to housing conditions and handling, rats were arbitrarily allocated to one of three groups: (i) infraorbital nerve chronic constriction injury (ION‐CCI) (*n* = 11) (Figure 1a), (ii) sham‐injury (Sham) (*n* = 6), or (iii) anesthesia only (Naïve) (*n* = 8). Each rat underwent sensory testing with von‐Frey filaments; although, in contrast to our previous study (Kang et al. 2024), due to imaging facility requirements, testing had to be conducted during the light period. Behavioral baselines to von‐Frey stimulation were established from testing 8 days and 1 day prior to surgery (Figure 1b). On the day of surgery, rats received either an ION‐CCI, a sham‐injury, or exposure to anesthesia alone (Naïve). Sensory testing resumed each week (+6, +13, +20, +27 days) for the duration of the experiment. Each rat completed a scanning session consisting of approximately 60 min of magnetic resonance imaging (MRI) and 60 min of computed (CT) and positron emission (PET) tomography image acquisition. This 120‐min scanning procedure was performed on each rat, 7 days prior to surgery (day −7), and again following either ION‐CCI, sham‐injury, or anesthesia alone, on days +2, +7, +14, and +28 (Figure 1c). Rats had to remain in the imaging facility for 48 h after PET imaging before being returned to their original home‐cages.

### 2.2 Imaging Procedures

All procedures were performed at the Alfred Research Alliance‐Monash Biomedical Imaging (ARA‐MBI) facility (Alfred Centre, Melbourne, Australia). Each rat was anesthetized by induction with isoflurane (5% in 100% oxygen), delivered in an airtight induction chamber. Anesthesia was maintained with 1.5%–3% isoflurane in 100% oxygen administered via a custom‐made facemask. Body temperature was maintained by a thermal blanket. Each rat was placed into a 9.4 Tesla Bruker MRI scanner for approximately 60 min during which a series of anatomical images were acquired. While still anesthetized, each rat was then transferred supine and headfirst into a Mediso NanoScan PET/CT (Mediso Ltd., Hungary) with the brain and heart in the field of view. [^18^F]‐SMBT1 was synthesized at the Peter MacCallum Cancer Centre with the radiochemical purity of [^18^F]‐SMBT1 always exceeding 95%, and the molar activity ~31,000 MBq/μmol. A maximum of 5 animals were imaged per imaging session.

At the start of the dynamic PET acquisition, rats were injected via the dorsal penile vein with a bolus of 0.1195 mCi to 1.1329 mCi of [^18^F]‐SMBT1, in volumes ranging from 0.03 mL to 0.80 mL. PET scans were acquired continuously for a total of 60 min with 1–3 coincidence mode, a coincidence time‐window of 5 ns and an energy window of 400–600 keV (Figure 1D). Following the PET scan, two CT scans were acquired over a period of 10 min. The first CT image was acquired using the same field of view as the PET and was used for attenuation correction (70 kVp at 700 μA, 480 projections per bed position, helical mode, scan length of 98.2 mm, 300 ms exposure time and 1:4 binning). The second CT image covered the head only and was used for co‐registration (70 kVp at 700 μA, 720 projections per bed position, semi‐circular single FoV mode, with medium zoom, scan length of 37.1 mm, 300 ms exposure time and 1:1 binning) (Figure 1e).

Prior to each scanning day, animal weight was recorded, and throughout scanning respiration and temperature were recorded to ensure animal safety and monitor for any physiological or autonomic changes relating to either the ION‐CCI injury model or tracer injection. These metrics were analyzed for between‐group interactions, summarized in Supporting Information and Table S7.

### 2.3 Surgical Procedures: Infraorbital Nerve Constriction Injury (ION‐CCI)

Rats were anesthetized by induction with isoflurane (5% in 100% oxygen), delivered in an airtight induction chamber. Surgical levels of anesthesia were maintained with 1.5%–2% isoflurane in 100% oxygen administered via a custom‐made facemask. Body temperature was maintained by a thermal blanket. ION‐CCI was performed as described by Vos et al. (1994). The following procedures were performed under ×3.5 magnification using Galilean Loupes (28050‐35 Fine Scientific Tools, Vancouver Canada). The fur on the midline above the nasal bones and between the eyes was shaved and the skin sterilized with povidone‐iodine. Once the blink reflex and pedal reflex were abolished, an incision was made approximately 12 mm long, and 2 mm medial to the supraorbital ridge of the right orbit, following the curvature of the frontal bone. The fascia and muscle were gently teased from the bone using blunt dissection until the contents of the orbit could be gently retracted laterally. Following retraction of the orbital contents, the ION was visualized approximately 8 mm deep within the orbit, lying within the infraorbital canal of the maxillary bone. Once visualized, 5 mm of the ION was gently freed from the surrounding connective tissue via blunt dissection. Two chromic gut ligatures (Chromic Gut 5‐0 sutures, #687G, Ethicon Inc.) were loosely tied around the ION using a square knot, tight enough to slightly compress the nerve, but not enough to occlude epineural circulation. The ligatures were spaced approximately 2 mm apart, and the ION was repositioned in the infraorbital canal of the maxillary bone. The incision was sutured closed with nylon sutures (Nylon Sutures 7‐0, #1696G, Ethicon Inc.). The incision site was cleaned, and antibiotic powder was dusted over the suture site. The rat was moved to a heated recovery cage and monitored closely until ambulatory and eating and drinking. Sham‐injury procedures were identical, but without any ligatures applied to the ION. Naïve, anesthesia‐only procedures were identical, with no surgical incision; the rats were maintained under isoflurane anesthesia for 40 mins.

### 2.4 PET Image Processing and Analysis

The raw PET list‐mode data was reconstructed using the Mediso software, Tera‐Tomo 3D reconstruction method, 2 iterations of a 3‐dimensional ordered subsets expectation maximization (3D OSEM) algorithm (6 subsets) and with a matrix size of 142 × 142 × 159, an isotropic voxel size of 0.6 mm and with the following corrections, attenuation, scatter, random and ^18^F‐decay. The length of the dynamic scan frames was 5 × 1 min, 5 × 2 min, 3 × 5 min, 3 × 10 min. Time‐activity‐curves were obtained to assess the kinetics of the [^18^F]‐SMBT‐1 radiotracer in the brain. The last 30 min, corresponding to the relatively stable transient equilibrium, were used to calculate the Standard Uptake Values (SUV_30–60 min_), which is SUV_30–60 min_ body weight [g/ml] = (Tissue activity/Decay corrected injected dose)*Weight*1000 (Figure S1). To account for the amount of radiotracer that crossed the blood–brain barrier, these SUV values were divided by the whole brain SUV for each rat to obtain the SUV ratio (SUVr_30–60 min_). PET images were then pre‐processed using the PMOD software (v4.404, PMOD Technologies), Statistical Parametric Mapping 12 (SPM12) and in‐house Matlab scripts (Matlab, Mathworks, R2023a). CT and SUV and SUVr images were co‐registered to the SIGMA Space atlas of the Wistar rat brain (Barrière et al. 2019). These co‐registered brain maps were then resliced at 0.3 × 0.3 × 0.3 mm voxel size and smoothed using a 0.9 mm full‐width‐at‐half‐maximum (FWHM) Gaussian kernel.

As [^18^F]‐SMBT‐1 is a novel radioligand for assessing astrocyte reactivity within the CNS, and is yet to be used in Sprague–Dawley rats for this purpose, tracer validation was performed by creating decay curves and calculating decay constants within the whole brain, as well as in four candidate reference regions: the cerebellum; the left and right thalamus proper; and the prelimbic cortex. In each region a 2.0 mm spherical volume‐of‐interest mask was created, centred on the SIGMA atlas parcel comprising these regions. From each dynamic scan frame prior to the generation of summed image sets, measures of tissue activity were extracted from each animal at each time point, converted into SUV, and exponential decay curves plotted for visual inspection between groups and time points for significance testing within‐ and between‐groups, as well as comparison with established literature on the decay constant of [^18^F]‐SMBT‐1 (Figure S1, Table S8). Additionally, coefficient of variation analyses were conducted using whole brain SUV values from the initial dynamic frame capturing tracer uptake, to assess within‐group variability. Single‐factor ANOVA analyses were further conducted using these same initial dynamic frame values, as well as the change in SUV between the initial and second dynamic frames to assess between‐group variability of initial tracer uptake and decay (Table S9).

To determine significant differences in [^18^F]‐SMBT‐1 uptake relating to orofacial neuropathic pain, a full factorial model was constructed using the contrast images at each time point of animals that underwent ION‐CCI. A single “time” factor was entered with five layers encoding the co‐registered, resliced, and smoothed SUVr brain images (days −7, +2, +7, +14, and +28). Since we hypothesized that the predominant changes in astrocyte reactivity would occur either during early or late time points relative to injury, two second‐level contrast images were generated, weighting either days 2 and 7 (“early”) or days 14 and 28 (“late”) as significantly greater in [^18^F]‐SMBT‐1 uptake relative to the alternate post‐injury time points and always significantly greater than baseline uptake (Figure 1f). Separate voxel‐by‐voxel second‐level, random effects analyses were conducted in both of these contrast images (*p* < 0.001, uncorrected for multiple comparisons, minimum cluster forming threshold of 20).

Volume‐of‐interest masks were created from each voxel forming significant cluster within either “early” or “late” analyses, with SUVr values then extracted from both the Sham and Naïve groups for post hoc determination of whether [^18^F]‐SMBT‐1 uptake significantly differed not only within injured animals across time, but between groups at any given time point. For these analyses, pre‐injury SUVr was normalized to a value of 1, with the denominator for normalization applied as a multiplier to each subsequent time point of each animal to give an identical baseline and assess change in binding as a relative percentage across time from pre‐injury. Following this process, Hedge's G effect size estimate analyses were conducted at either day +28 (for late change clusters) or day +2 (for early change clusters) to delineate within each brain region whether small, medium, or large differences in percentage change in SUVr from baseline were present between ION‐CCI and naïve or sham animal groups. Moreover, a mean (±SEM) was also calculated and plotted for each group, with paired *t*‐test analyses conducted in ION‐CCI animals between day +2 and +28 SUVr changes, and separate two‐sample *t*‐tests conducted at day +2 and day +28 between the three groups.

### 2.5 Euthanasia and Perfusion

Forty‐eight hours following the final imaging session, rats were anesthetized briefly with 5% isoflurane mixed in oxygen 1.5 L/min followed by deep, terminal pentobarbitone anesthesia (130 mg/kg, intraperitoneal). Each rat was then perfused trans‐cardially with 400 mL of ice cold heparinised 0.9% (w/v) saline, followed by 400 mL of 4% paraformaldehyde in sodium acetate‐borate buffer (pH 9.6, 4°C) (PFA). The brain of each rat was isolated and removed and placed in PFA for 2 h at room temperature, then cryoprotected in 10% (w/v) sucrose in 0.1 M phosphate buffered saline, pH 7.4 (PBS) and stored at 4°C until processing. In addition, the right infraorbital nerve (ION), and its trigeminal ganglion (TG) were also collected in PFA, cryoprotected and stored for reference.

### 2.6 Histological Verification

The brain of each rat was sectioned using a cryostat (LEICA CM1950, Germany). The brainstem was isolated with a coronal cut caudally at approximately the C3 level of the cervical spinal cord, and rostrally at the level of the inferior colliculus (−9.24 mm bregma). The remaining brain tissue was cut into two at the level of the anterior commissure (−0.12 mm bregma^34^). Free‐floating coronal sections of the brain were cut at 40 μm and stored in antifreeze at −20°C until processed. In addition, the right ION and its corresponding TG of three rats (exemplars of ION‐CCI, Sham and Naïve) were cut (14 μm) and collected onto gelatinised (2% w/v) slides and stored at −20°C until processing.

### 2.7 Processing and Imaging of Brain Sections

Free‐floating sections were washed in serial dilutions of ethanol (100%, 90% and 70% (v/v)) for 2‐min each, before being rinsed in dH_2_O. Sections were then washed in phosphate buffered saline, pH 7.4 (PBS) and then blocked in 5% (v/v) normal horse serum (NHS) in PBS for 30‐min at room temperature. Sections were co‐incubated for 16‐h at 4°C with rabbit anti‐MAO‐B [EPR24131‐79] (1:250, Abcam, Melbourne, Vic, Australia, Cat# ab259928; batch GR3379189‐4) and mouse anti‐GFAP (1:5000, Sigma‐Aldrich, Australia Cat# G3893, RRID:AB_477010) in PBS containing 5% (v/v) NHS. Sections were then washed in PBS and co‐incubated for 2‐h in Alexa‐488 conjugated donkey anti‐mouse (1:500, RRID:AB_2340846, Jackson, West Grove, PA, USA) and Cy3 conjugated donkey anti‐rabbit (1:500, RRID:AB_2307443, Jackson, West Grove, PA, USA). Slides were washed in PBS and then incubated for 20‐mins with DAPI (ThermoFisher Scientific, RRID:AB_2307445) in PBS. Sections were mounted onto gelatinised (2%) glass slides and cover slipped with Prolong Gold Antifade (P36931, Invitrogen) mounting medium and stored at 4°C until microscopy. Slides were imaged using a Nikon C2 confocal microscope. Brain sections containing the regions of interest defined by the late phase [^18^F]‐SMBT1 standardized uptake value ratio changes were identified using cytoarchitectural features with reference to a standard rat brain atlas (Paxinos 2009). Images of MAO‐B and GFAP immunoreactivity were captured at x200 magnification ensuring that the region of interest defined by the late phase [^18^F]‐SMBT1 was at the centre of the image. These images were then subjected to analysis described below.

### 2.8 Quantitative Analysis of Brain MAO‐B Immunoreactivity

Integrated density analysis of MAO‐B immunoreactivity (MAO‐B‐IR) was performed for each region of interest using FIJI (Image J). The percentage area of MAO‐B‐IR or “integrated density” per image was determined using<*threshold*>, <*create mask*> and <*analyze* particles> functions. The integrated densities at each location for rats in each group ION‐CCI, Sham and Naïve were compared using estimation statistics, as encouraged by Calin‐Jageman and Cumming (Calin‐Jageman and Cumming 2019) and Bernard (Bernard 2019), and previously used in our lab Boorman and Keay (2023). Estimation statistics provides both an estimate of the effect size and an uncertainty interval, expressed here as the 95% confidence interval. Estimation statistics were calculated using the web application built by Hung Nguyen (estimationstats.com), which uses the Python code developed by Ho et al. (Ho et al. 2019). Effect sizes (Cohen's d) and their 95% confidence intervals were calculated using bias‐corrected and accelerated bootstrap resampling (5000 bootstrap samples per test). When reporting these results, effect sizes between 0.2 and 0.5 were considered “small”, effect sizes between 0.5 and 0.8 were considered “moderate”, and effect sizes above 0.8 considered “large”.

### 2.9 Colocalization Analysis

We evaluated the colocalization of MAO‐B and GFAP using FIJI (Image J). A mask of MAO‐B‐IR/GFAP‐IR images was first separated using the <split channel> function; following this, a mask of each image was created using <threshold> and <create mask> functions. The degree of co‐localisation was determined using the plugin <JACoP> (Bolte and Cordelières 2006), and to perform a correlation‐based colocalization using Mander's overlap co‐efficient (Manders et al. 1992). The Mander's overlap co‐efficient was defined as (1) MAO‐B‐IR/GFAP‐IR (M/G) as the ratio of the “summed intensities of pixels from the MAO‐B‐IR image for which the intensity in the GFAP‐IR channel is above zero” to the “total intensity in the MAO‐B‐IR channel”; and (2) GFAP‐IR/MAOB‐IR (G/M) is defined as the converse. A single outlier data point was excluded from the MAO‐B‐IR analysis as the integrated density was greater than 3 standard deviations from the mean.

### 2.10 Processing and Imaging of ION and TG

To illustrate MAO‐B expression in the ION and TG sections, 3 rats were immersed in xylene (534056‐4 L Sigma‐Aldrich, Australia); then an identical immunohistochemical procedure to that used to identify MAO‐B and GFAP in brain sections was used (see above). Slides were imaged using a Nikon C2 confocal microscope. The large image function was used to “stitch” multiple 100× magnification images to allow the visualization of the entire infraorbital nerve including ligated areas and trigeminal ganglia. Representative 400× magnification images or z‐stack images were captured at 400× magnification to visualize MAO‐B and GFAP‐IR.

## 3 Results

Results are presented from longitudinal von‐Frey behavioral testing and imaging analyses evaluating the hypotheses of discrete e*arly onset* injury‐evoked changes in [^18^F]‐SMBT‐1 binding and *late onset* injury‐evoked changes in [^18^F]‐SMBT‐1 binding (Figure 1f). Finally, data from post‐mortem analyses of MAO‐B‐IR in the regions of interest identified by late onset [^18^F]‐SMBT‐1 binding are presented, as well as their relationship to GFAP expression.

### 3.1 Behavioral Responses to Mechanical (von Frey) Stimulation

**FIGURE 2:**
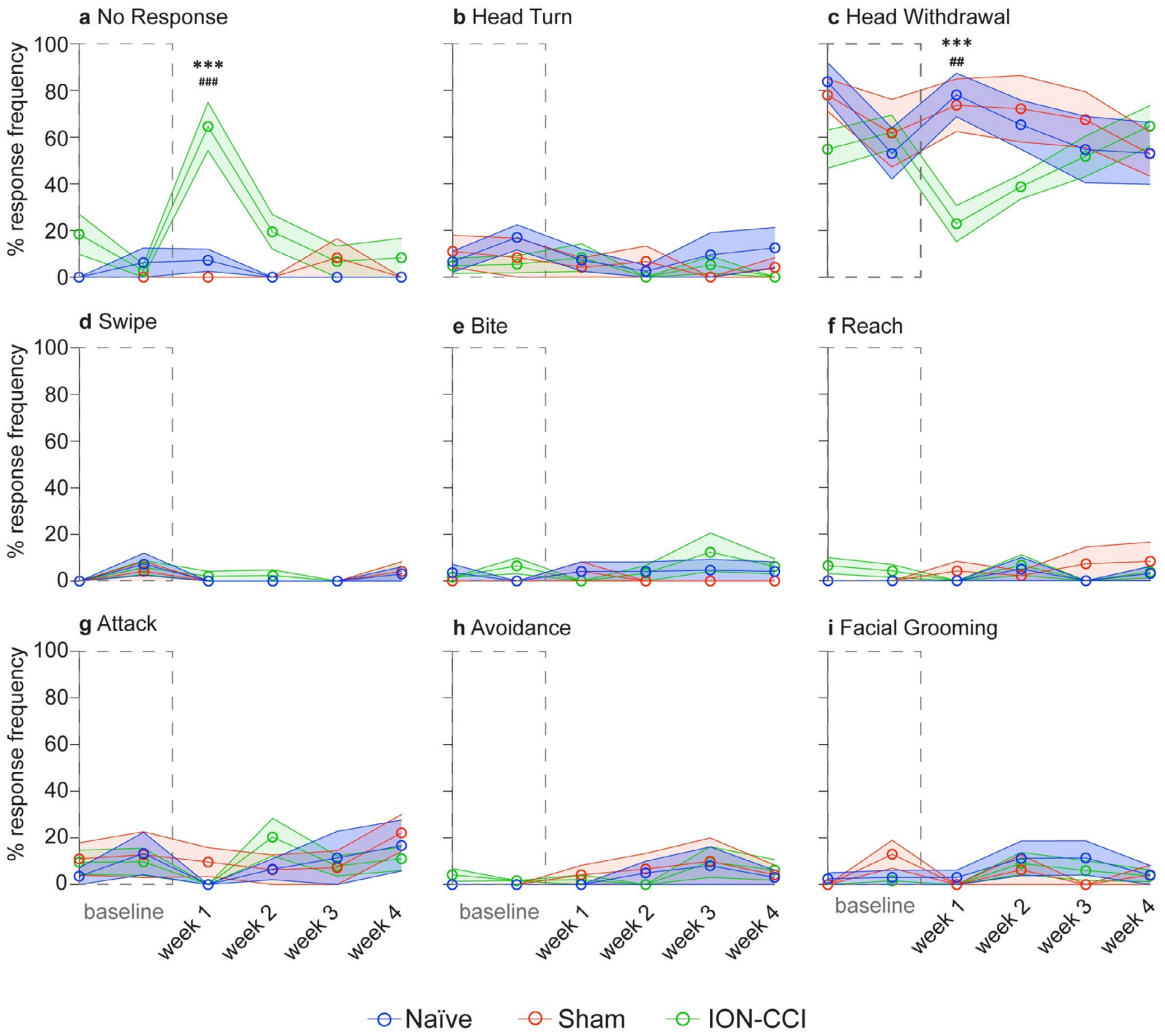
Behavioral response to mechanical stimulation of the facial skin in NaÏve, Sham and ION‐CCI male rats. The percentage response frequency from experimental stimulations is categorized as: (a) No Response; (b) Head Turn; (c) Head withdrawal; (d) Swipe; (e) Bite; (f) Reach; (g) Attack; (h) Avoidance or (i) Facial grooming. The changes in behavioral responses of NaÏve (blue), Sham‐injury (red) and infra‐orbital nerve chronic constriction injury animals (ION‐CCI: Green) are depicted over a baseline period of a total of 8 days prior to surgical procedures, and a post‐surgical period of 4 weeks. Significant differences are represented as *p < 0.001 when ION‐CCI rats are compared to NaÏve rats or ^##^ *p* < 0.01, ^###^ *p* < 0.001when ION‐CCI rats are compared to Sham rats. Mixed‐effect analysis, with Tukey multiple comparisons *post hoc* test. Error bars represent ±SEM (standard error of the mean).

Analysis of the video recordings of the behavioral responses of Naïve rats to von‐Frey stimulation of the area of the facial skin innervated by the ION revealed a more restricted range of behaviors than we have previously reported (Kang et al. 2024). The most frequently observed behavior was head withdrawal followed by biting, attacking and head turns. The behaviors seldom observed were reaching, swiping, attack, avoidance and facial grooming (Figure 2). In the majority of cases, von‐Frey stimulation evokes only a single behavioral reaction, and in ~10% of stimuli head withdrawal followed by biting or attacking is the common responses. To contrast, stimulation of the same facial region in ION‐CCI rats during the early post‐injury period (week 1–2) did not evoke a behavioral response as indicated by a reduction in head withdrawals [F_2,133_ = 6.571; *p* = 0.0019], whereas in later tests (week 3–4) the von Frey stimulation almost exclusively evoked single behavioral responses which were either head withdrawals or attack. This pattern of injury related behavioral reactions is almost identical to our earlier report (Kang et al. 2024). In sham‐injured rats, the responses to mechanical stimulation of the facial skin evoked behaviors that were similar to those evoked in the Naïve group and again was almost identical to findings reported in our earlier report (Kang et al. 2024).

### 3.2 Early Onset Injury‐Evoked Changes in ^18^F‐SMBT‐1 Binding: (Early Phase Model)

**FIGURE 3:**
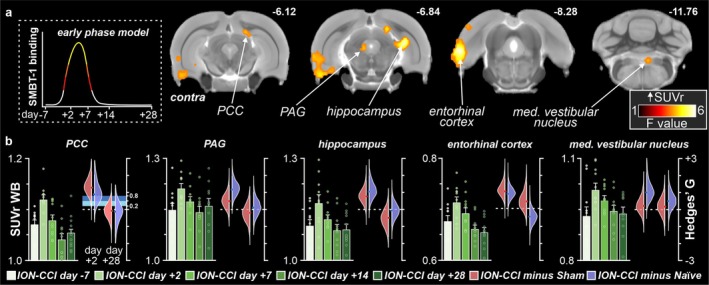
Early phase [^18^F]‐SMBT1 standardized uptake value ratio changes in the rat brain following infraorbital nerve constriction. Voxel‐by‐voxel Repeated measured factorial analysis were conducted using whole brain standardized uptake value ratio (SUVr) images of animals that underwent infraorbital nerve chronic constriction injury (ION‐CCI) (*n* = 11). (a) Early and transient [^18^F]‐SMBT‐1 binding increases that began immediately following ION‐CCI were identified in the midbrain periaqueductal gray (PAG), the ipsilateral hippocampus, posterior cingulate cortex (PCC), and the medial vestibular nuclei of the medulla. (b) SUVr values were extracted from ION‐CCI animals at each timepoint to visualize these early changes, and Hedge's G effect size differences calculated at days +2 and +28 between groups through volume‐of‐interest extraction of these clusters from Naïve and Sham cohorts. Red violin plots display the effect size and confidence interval differences between ION‐CCI and Sham groups, and blue between ION‐CCI and Naïve groups. Binding increases are indicated by the hot color scale overlaid onto a T2‐weighted anatomical template. Slice locations relative to Bregma are indicated at the top right of each coronal slice. contra = contralateral.

During early injury stages (days +2 and +7), significantly increased [^18^F]‐SMBT‐1 uptake was observed in several sites at the level of the midbrain. ION‐CCI evoked increased uptake of [^18^F]‐SMBT‐1 in the lateral column of the periaqueductal gray (lPAG) contralateral to the nerve injury, as well as the hippocampus and posterior cingulate cortex (PCC) ipsilateral to the ION‐CCI (Figure 3a, Tables [Link], [Link]). A significant difference was observed between day +2 and day +28 post‐ION‐CCI as revealed by paired *t*‐tests, with a significantly greater uptake at day +2 relative to day +28 (mean ± SEM SUVr % change Δpd7 lPAG: day +2 = 6.65 ± 1.73, day +28 = 1.29 ± 2.41, *p* = 0.03; hippocampus: day +2 = 6.95 ± 1.34, day +28 = −0.95 ± 1.65, *p* = 0.01; PCC: day +2 = 4.83 ± 0.67, day +28 = −1.50 ± 1.11, *p* = 0.001). Two additional sites were also identified: the entorhinal cortex contralateral to ION‐CCI; and the medial vestibular nucleus ipsilateral to ION‐CCI, each of which displayed significantly increased binding in the early phase of injury. Post hoc paired *t*‐tests revealed (mean ± SEM SUVr % change Δpd7 entorhinal cortex: day +2 = 6.14 ± 1.70, day +28 = −3.06 ± 1.68, *p* = 0.003; medial vestibular nucleus: day +2 = 7.22 ± 2.59, day +28 = 1.01 ± 2.43, *p* < 0.001) (Table S1).

Additionally, Hedge's G analyses revealed predominantly large effect sizes in the change in SUVr relative to baseline values between ION‐CCI animals and both Naïve and Sham groups within each of these four sites at day +2, which had reduced to either small or medium effect size differences by day +28. Two days following the ION‐CCI, within the lPAG a large effect size was observed between ION‐CCI and Naïve group (Hedge's G = 1.18 [95% CI 0.17, 1.95]); and a small effect size between ION‐CCI and Sham (Hedge's G = 0.487 [95% CI −0.44, 1.39]). In both the hippocampus and medial vestibular nucleus, a large effect size was observed between ION‐CCI and Naïve (Hippocampus: Hedge's G = 1.06 [95% CI 0.02, 1.97]; Medial vestibular nucleus: Hedge's G = 1.00 [95% CI 0.06, 2.17]), and a medium effect between ION‐CCI and Sham (Hippocampus: Hedge's G = 0.76 [95% CI −0.10, 1.68]; Medial vestibular nucleus: Hedge's G = 0.21 [95% CI −0.67, 1.28]). In the PCC and entorhinal cortex, large effect sizes were observed between ION‐CCI and both Naïve (PCC: Hedge's G = 0.88 [95% CI −0.55, 2.03]; entorhinal cortex: Hedge's G = 0.91 [95% CI 0.16, 1.57]), and ION‐CCI and Sham (PCC: Hedge's G = 1.28 [95% CI 0.12, 2.22]; entorhinal cortex: Hedge's G = 1.06 [95% CI 0.18, 1.79]) (Figure 3b). Post hoc two sample *t*‐test analyses performed at each time point between groups were consistent with regions demonstrating the largest Hedge's G effect size differences (Figure S2). Volume of interest analyses for each of these clusters from both Naïve and Sham cohorts revealed no significant effect over time in any single cluster in either cohort (Tables S2 and S3).

### 3.3 Late Onset Injury‐Evoked Changes in [ ^18^F]‐SMBT‐1 Binding: (Late Phase Model)

**FIGURE 4:**
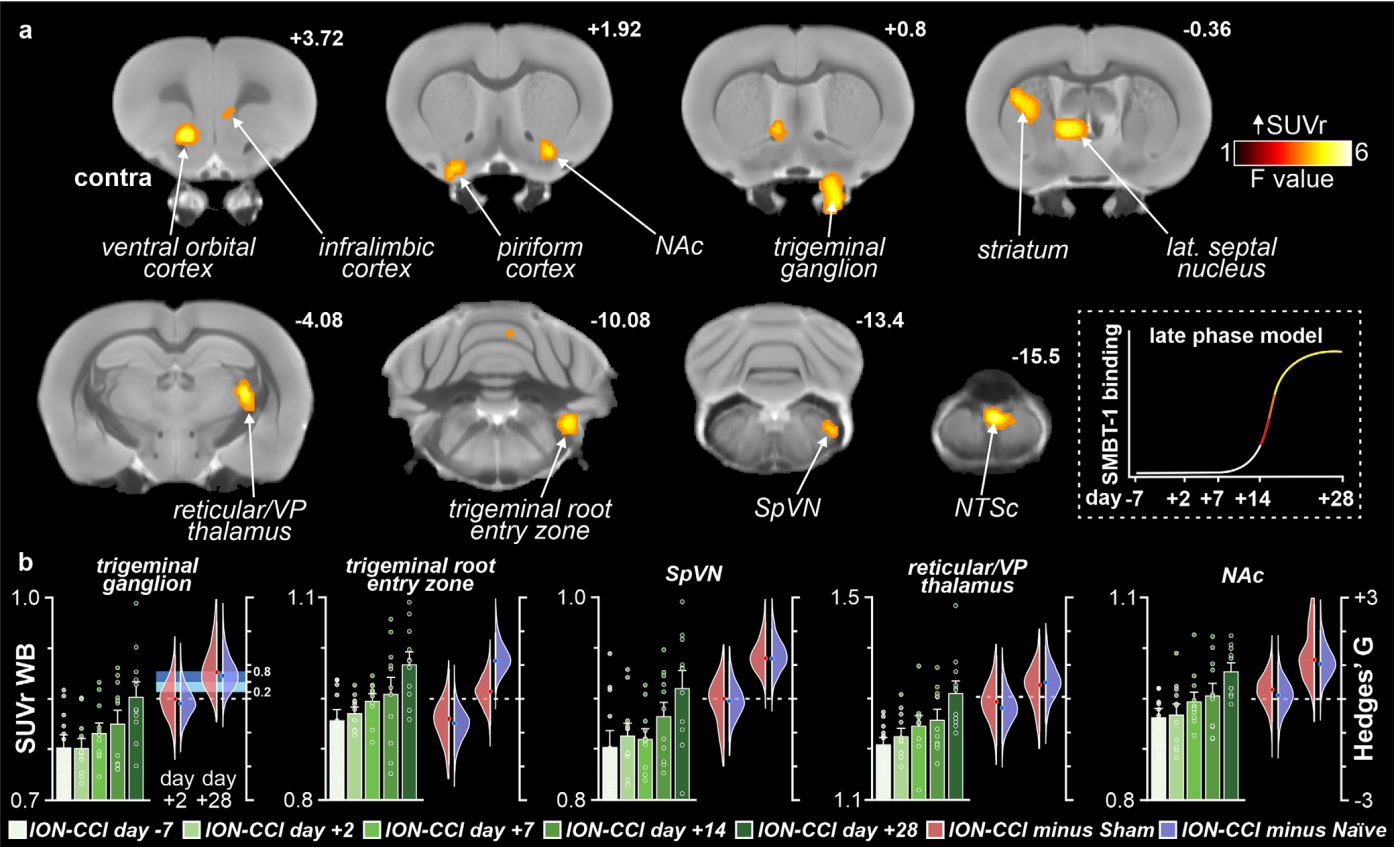
Late phase [^18^F]‐SMBT1 standardized uptake value ratio changes following ION‐CCI. Voxel‐by‐voxel, repeated measures factorial analysis was conducted using whole brain standardized uptake value ratio (SUVr) images of rats that underwent ION‐CCI (*n* = 11). (a) Late onset binding increases occurred in the commissural nucleus of the nucleus tractus solitarius (NTSc), the ipsilateral trigeminal ganglion and trigeminal root entry zone, the spinal trigeminal nucleus (SpVN), the reticular and ventral posterior thalamic nuclei (VP), lateral nucleus accumbens (NAc) shell, contralateral ventral orbital and piriform cortices, the lateral septal nuclei, and in the lateral aspect of the dorsal striatum. (b) SUVr values were extracted from ION‐CCI animals at each timepoint to visualize these late changes, and Hedge's G effect size differences calculated at days +2 and +28 between groups through volume‐of‐interest extraction of these clusters from naïve and sham animal cohorts. Red violin plots display the effect size and confidence interval differences between ION‐CCI and sham groups, and blue between ION‐CCI and naïve groups. Binding increases are indicated by the hot color scale overlaid onto a T2‐weighted anatomical template. Slice locations relative to Bregma are indicated at the top right of each coronal slice. Contra = Contralateral.

Regional [^18^F]‐SMBT‐1 binding in ION‐CCI rats in late injury stages (day +14 and day +28) showed a different pattern to that seen in the *early* phase model. These included, selectively on the right, the trigeminal ganglion, the trigeminal root entry zone (TREZ), the SpVN, and the ventral posterior thalamus (VP), each a critical locus of the classical ascending sensory pathway for orofacial pain (Figure 4a, Tables [Link], [Link]). While post hoc tests did not reveal differences between baseline and day +2 binding between ION‐CCI, Sham, and Naïve rats, paired *t*‐tests revealed that all clusters in ION‐CCI rats showed significantly greater SUVr percentage changes from pre‐injury levels at day +28, when compared with day +2 (mean ± SEM SUVr % change Δpd7 trigeminal ganglion: day +2 = 0.33 ± 2.46, day +28 = 9.92 ± 2.43, *p* < 0.001; TREZ: day +2 = 1.37 ± 1.40, day +28 = 9.33 ± 2.68, *p* = 0.007; SpVN: day +2 = 1.48 ± 1.07, day +28 = 7.07 ± 2.11, *p* = 0.02; VP: day +2 = 1.01 ± 0.01, day +28 = 1.08 ± 0.02, *p* = 0.009) (Figure 4b).

Contralateral to the ION‐CCI we also identified increased uptake in the commissural nucleus of the nucleus tractus solitarius (NTSc), the lateral aspect of the dorsal striatum (DS), the ventral part of the lateral septal nuclei, the piriform cortex and the ventral orbital cortex. Ipsilateral to the ION‐CCI we identified increased uptake in the infralimbic cortex, the lateral nucleus accumbens shell region (NAc) (Figure 4a). In each region, ION‐CCI rats had greater [^18^F]‐SMBT‐1 uptake at day +28 compared to day +2 (mean ± SEM SUVr % change Δpd7 NTSc: day +2 = 3.04 ± 1.62, day +28 = 7.06 ± 2.60, *p* = 0.01; DS: day +2 = −2.52 ± 2.08, day +28 = 8.86 ± 3.42, *p* = 0.005; lateral septal nuclei: day +2 = 3.27 ± 1.48, day +28 = 10.53 ± 2.42, *p* = 0.02; piriform cortex: day +2 = 1.62 ± 1.66, day +28 = 8.37 ± 1.77, *p* = 0.01; ventral orbital cortex: day +2 = 2.42 ± 2.09, day +28 = 9.33 ± 2.20, *p* = 0.004; infralimbic cortex: day +2 = 4.85 ± 3.70, day +28 = 12.40 ± 3.50, *p* = 0.04; NAc: day +2 = 0.54 ± 1.63, day +28 = 7.51 ± 1.49, *p* = 0.001) (Figure 4b).

Hedge's G analyses revealed a mix of effect size changes in SUVr between groups at day +28. Within both the SpVN and NAc, large effect sizes were observed between ION‐CCI and both naïve (SpVN: Hedge's G = 1.20 [95% CI 0.09, 2.07]; NAc: Hedge's G = 1.03 [95% CI 0.41, 2.01]), and Sham (SpVN: Hedge's G = 1.22 [95% CI 0.33, 2.27]; NAc: Hedge's G = 1.15 [95% CI −0.66, 2.77]) animals. Dissimilarly, the DS and lateral septal nuclei demonstrated medium effect sizes between ION‐CCI and Naïve rats (DS: Hedge's G = 0.72 [95% CI −0.11, 1.32]; lateral septal nuclei: Hedge's G = 0.62 [95% CI −0.30, 1.47]), and large effect sizes between ION‐CCI and Sham rats (DS: Hedge's G = 0.89 [95% CI 0.09, 1.47]; lateral septal nuclei: Hedge's G = 1.18 [95% CI 0.49, 1.92]). In the TREZ, we observed large effect size differences between ION‐CCI and Naïve rats (Hedge's G = 1.11 [95% CI 0.18, 1.94]) and a small effect size between ION‐CCI and Sham rats (Hedge's G = 0.20 [95% CI −0.78, 1.14]). Contrasting the TREZ, the trigeminal ganglion demonstrated medium effect size differences between the ION‐CCI and both Naïve (Hedge's G = 0.67 [95% CI −0.30, 1.73]) and Sham (Hedge's G = 0.78 [95% CI −0.59, 2.25]) rats. In the piriform cortex, a small effect size difference was observed between ION‐CCI and Naïve rats (Hedge's G = 0.244 [95% CI −0.69, 1.25]), and a large effect size between ION‐CCI and Sham rats (Hedge's G = 1.03 [95% CI −0.42, 2.33]). The infralimbic cortex demonstrated a medium effect size difference at between ION‐CCI and Naïve (Hedge's G = 0.61 [95% CI −0.36, 1.54]), and a small effect size difference between ION‐CCI and Sham (Hedge's G = 0.37 [95% CI −0.47, 1.29]) rats. Finally, within each of the VP, ventral orbital cortex, and NTSc, only small effect size differences between each of the ION‐CCI and Naïve (VP: Hedge's G = 0.48 [95% CI −0.72, 1.53]; ventral orbital: Hedge's G = 0.39 [95% CI −0.87, 1.40]; NTSc: Hedge's G = 0.29 [95% CI −0.64, 1.20]) and ION‐CCI and Sham (VP: Hedge's G = 0.39 [95% CI −0.96, 1.82]; ventral orbital: Hedge's G = 0.31 [95% CI −0.92, 1.43]; NTSc: Hedge's G = 0.41 [95% CI −1.00, 1.44]) were identified. No between‐group differences were identified for the NTSc, the piriform cortex, the ventral orbital cortex, or the infralimbic cortex. Saving each of these clusters as volume‐of‐interest masks and extracting SUVr values from Naïve or Sham cohorts did not reveal significant effects of time in any cluster, in either cohort (Tables S2 and S3).

### 3.4 Post‐Mortem MAO‐B Immunohistochemical Analysis

**FIGURE 5:**
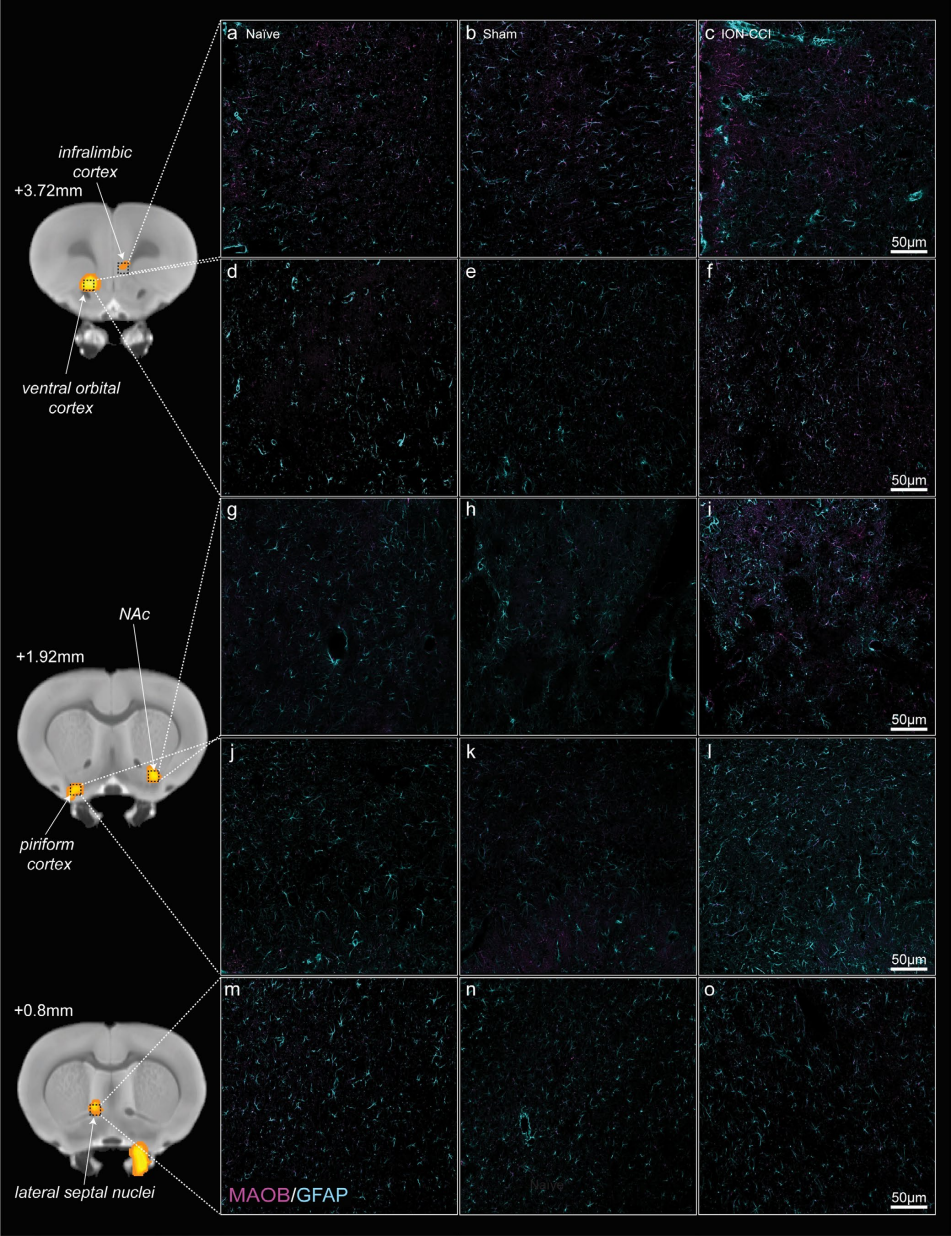
Representative monoamine oxidase‐B and glial fibrillary acidic protein immunoreactivity in the brain. 200× magnification photomicrographs depict monoamine oxidase‐B immunoreactivity (MAOB) (magenta) and glial fibrillary acidic protein immunoreactivity (GFAP) (turquoise) in Naïve (left column), Sham (middle column) and ION‐CCI (right column). (a–c) Infralimbic cortex; (d–f) ventral orbital cortex; (g–i) Nucleus Accumbens (NAc), lateral shell region; (j–l) piriform cortex; (m–o) lateral septal nuclei. Far left column shows [^18^F]‐SMBT‐1 binding increases indicated by the hot color scale overlaid onto a T2‐weighted anatomical template. Slice locations relative to Bregma in mm are indicated at the top left of each coronal slice according to Paxinos and Watson (Paxinos 2009). Scalebars represent 50 μm.

**FIGURE 6:**
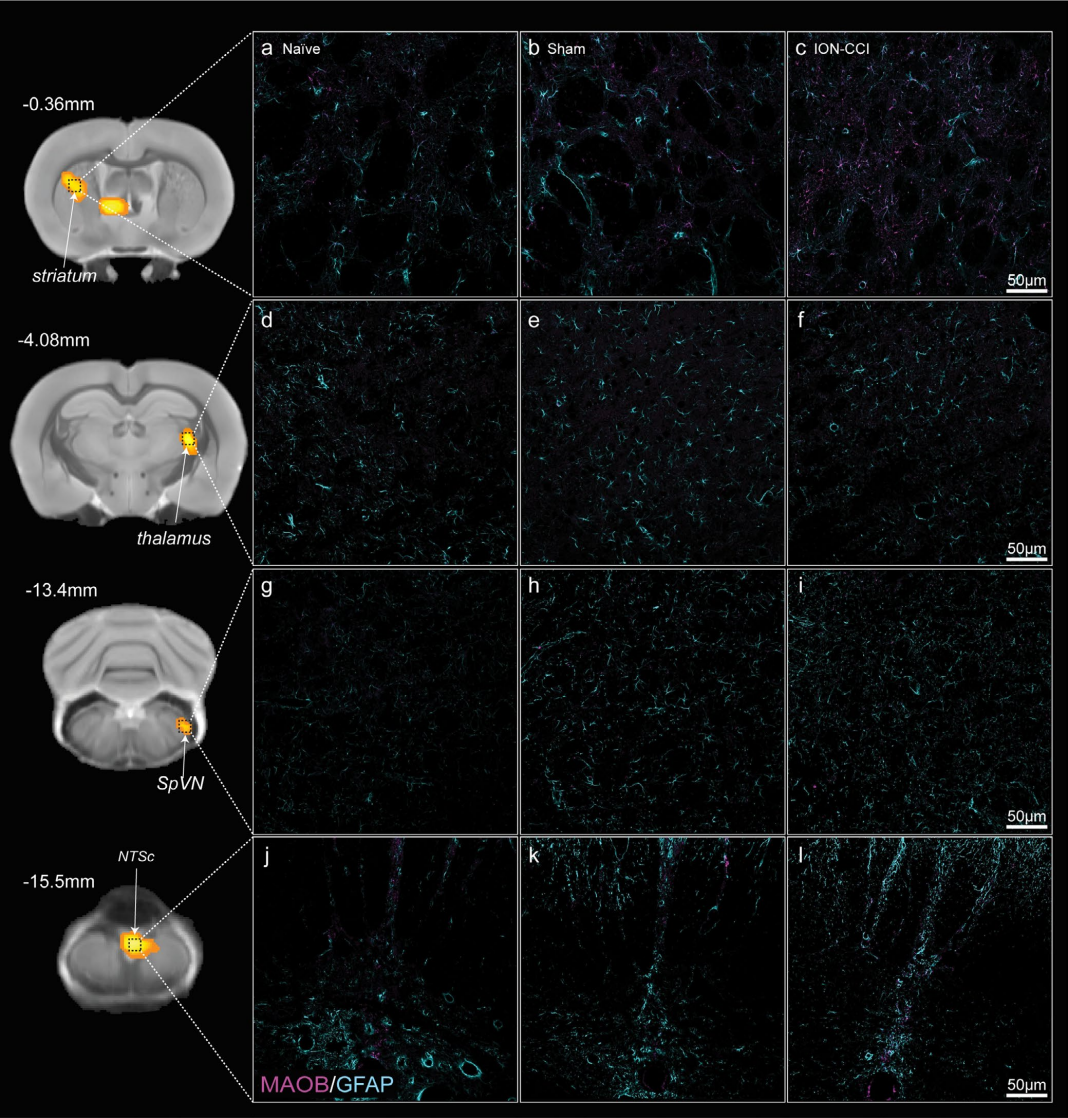
Representative monoamine oxidase‐B and glial fibrillary acidic protein immunoreactivity in the brain. 200× magnification photomicrographs depict monoamine oxidase‐B immunoreactivity (MAOB) (magenta) and glial fibrillary acidic protein immunoreactivity (GFAP) (turquoise) in Naïve (left column), Sham (middle column) and ION‐CCI (right column). (a–c) striatum; (d–f) thalamus; (g–i) Spinal trigeminal nucleus (SpVN); (j–l) commissural nucleus of the nucleus of the solitary tract (NTSc). Far left column shows [^18^F]‐SMBT‐1 binding increases indicated by the hot color scale overlaid onto a T2‐weighted anatomical template. Slice locations relative to Bregma in mm are indicated at the top left of each coronal slice according to Paxinos and Watson (Paxinos 2009). Scalebars represent 50 μm.

**FIGURE 7:**
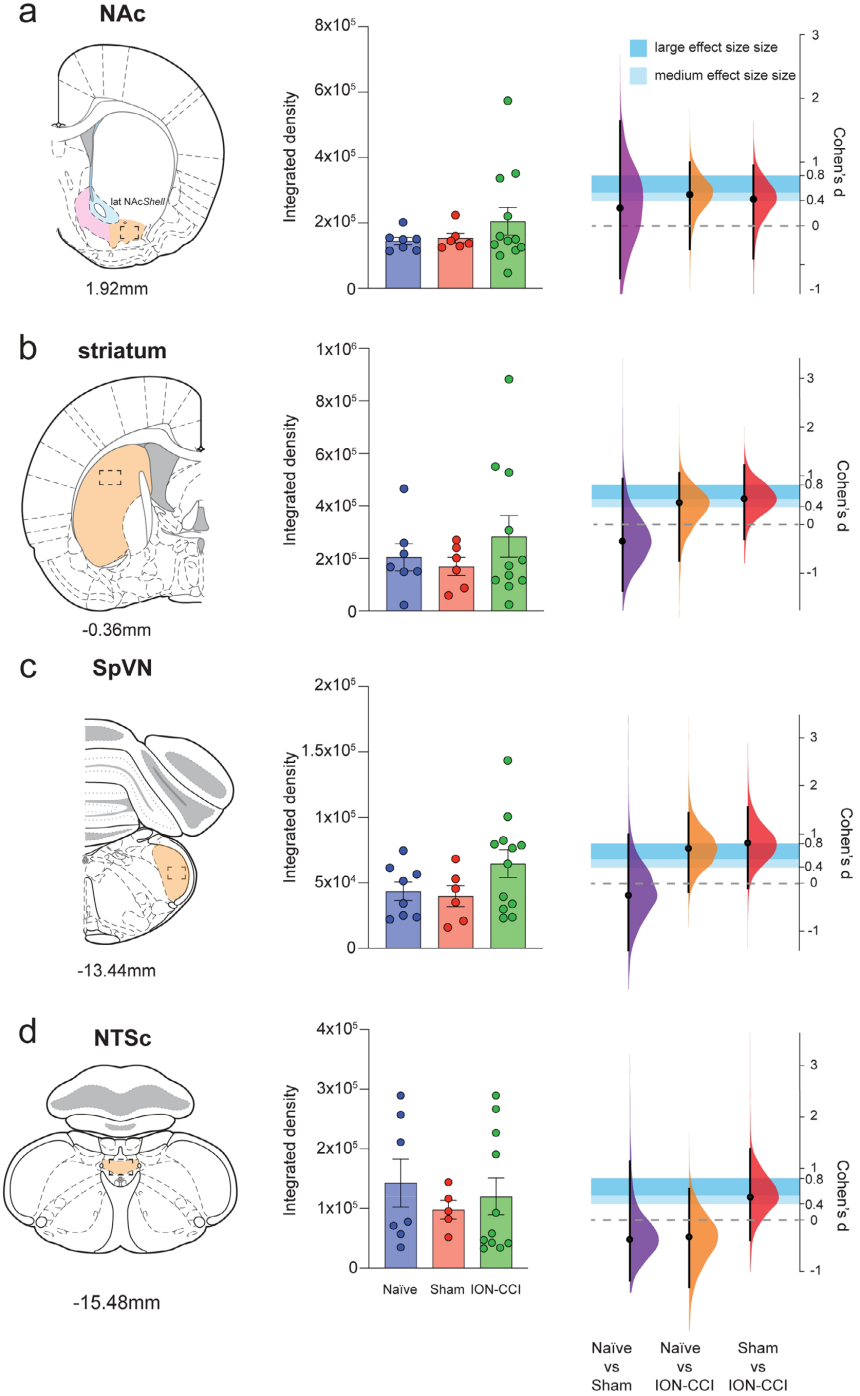
Monoamine oxidase‐B immunoreactivity. The integrated density of Monoamine oxidase‐B immunoreactivity with a medium or large effect size of ION‐CCI is presented in the figure. The regions of interest identified from the late phase [^18^F]‐SMBT1 standardized uptake value ratio changes in the rat brain were identified using cytoarchitectural features with references to the rat brain atlas Paxinos and Watson (Paxinos 2009). (a–d) depict the regions analyzed highlighted in orange. The location of the photomicrograph taken at x200 magnification for integrated density analysis is denoted by a dashed box. (e–f) histogram of the integrated density of MAOB immunoreactivity for NaÏve, Sham or ION‐CCI rats. The effect sizes (paired Cohen's d) between experimental conditions are shown on the Cummings estimation plots, with mean differences plotted as a bootstrap sampling distribution. Mean differences are depicted as dots; 95% confidence intervals are indicated by the ends of the vertical error bars. Medium effects are represented by light blue background and large effects are represented by blue background. The distance from bregma is shown under each representative coronal rat brain atlas section. Experimental conditions NaÏve (blue), Sham (red), ION‐CCI (green).

Photomicrographs showing MAO‐B immunoreactivity in the regions of interest identified as having late onset injury‐evoked binding changes in [^18^F]‐SMBT‐1 in each experimental group are shown in Figures 5 and 6 and comparisons between integrated densities of MAO‐B‐IR for each group are summarized in Table S4. In the infralimbic cortex, MAO‐B‐IR was present in all three groups (Figure 5a–c). Sham rats had greater MAO‐B‐IR compared with Naïve rats (Cohen's d: 0.609 [95% CI −0.812, 1.81]) (Figure 5 and Table S5). In the ventral orbital cortex MAO‐B‐IR was present in all three groups, and no differences were detected between them (Figure 5d–f and Table S5). In the shell region of the lateral NAc, while all three groups showed evidence of MAO‐B‐IR, the ION‐CCI group exhibited the highest density of MAO‐B compared with both Naïve (Cohen's d: 0.505 [95% CI −0.376, 1.03]; Figure 7a) and to a lesser degree the Shams (Cohen's d: 0.419 [95% CI −0.516, 0.947]; Figure 7a), the MAO‐B‐IR appeared with a “patch‐like” distribution (Figure 5g–i and Table S5). In both the piriform cortex and the lateral septal nuclei there were no differences detected in MAO‐B‐IR between the three groups (Figure 5j–o and Table S5). In the dorsal striatum, all three groups showed evidence of MAO‐B‐IR, and the ION‐CCI group exhibited a higher density of MAO‐B compared with the Shams (Cohen's d = 0.52 [95% CI −0.283, 1.19]; Figure 6a–c and Figure 7b). In the ventral posterior thalamus, MAO‐B‐IR was present in all three groups, and no differences were detected between them (Figure 6d–f and Table S5). Following ION‐CCI, MAO‐B‐IR labeled the SpVN strongly when compared with both Naïve (Cohen's d = 0.677 [95% CI −0.187, 1.45]; Figure 7c) and Sham rats (Cohen's d = 0.774 [95% CI −0.105, 1.57]; Figure 7c), we noted that the MAO‐B appeared to be in close association with small vessels supplying the medullary dorsal horn and the dorsolateral medulla (Figure 6g–i). In the NTSc, MAO‐B‐IR was present at the level of the commissural subnucleus, it was strongest in ION‐CCI rats when compared to Sham rats (Cohen's d = 0.486 [95% CI −0.393, 1.38]; Figure 6j–l and Figure 7d).

**FIGURE 8:**
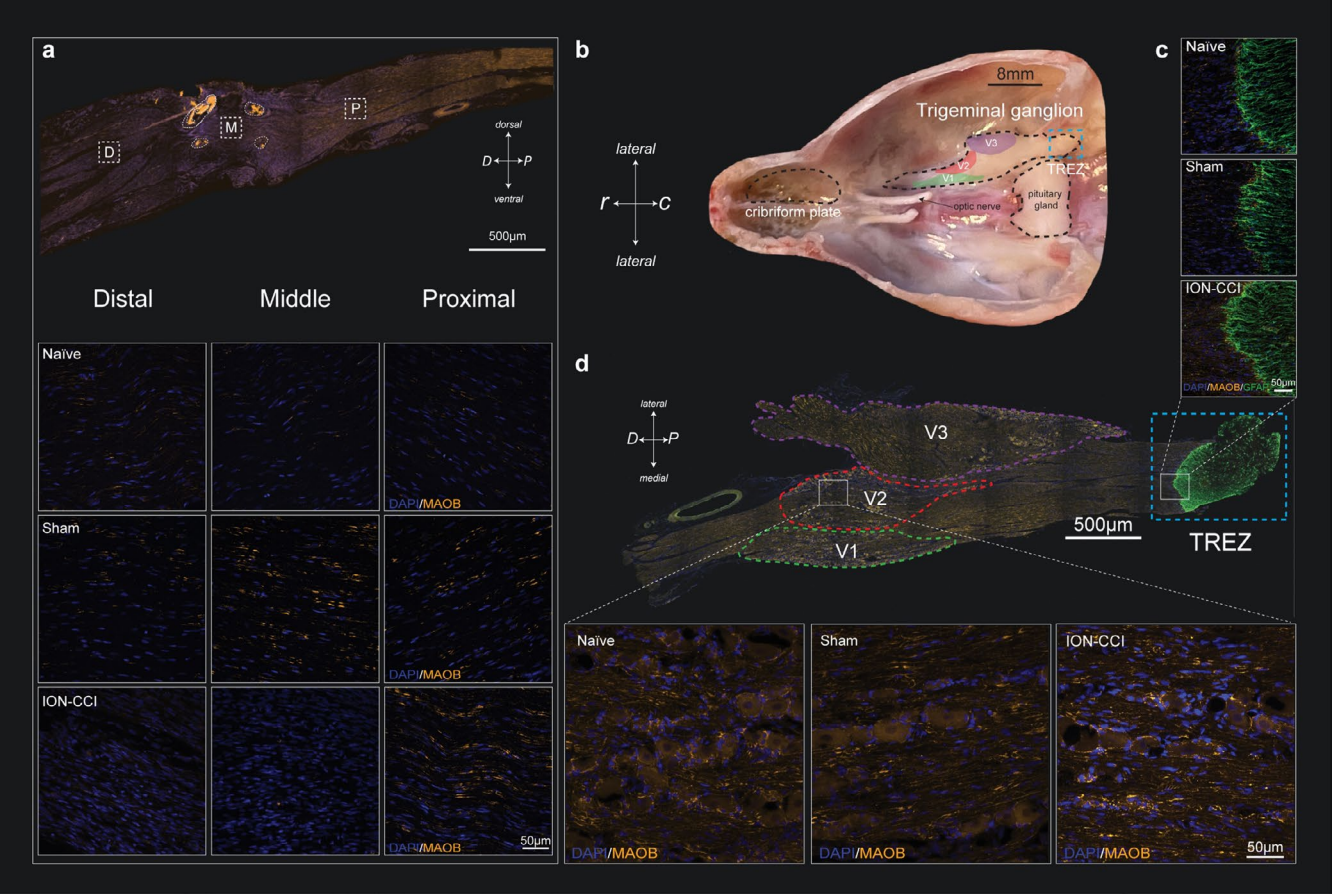
Representative monoamine oxidase‐B immunoreactivity in the peripheral trigeminal nervous system. (a) Stitched 100× magnification photomicrograph of monoamine oxidase‐B immunoreactivity (MAOB) (orange) and DAPI (blue) in a ligated (white dotted lines) infraorbital nerve. Photomicrographs taken for representative images located distal (D) to ligations, middle (M; between ligations) and proximal (P) are shown in white dashed boxes. Photomicrographs at 400× magnification of MAOB and DAPI in the Naïve, Sham and ION‐CCI rats. (b) Photo of the intracranial skull anatomy of a rat with the location of the trigeminal ganglion and its corresponding ophthalmic (V1, purple), maxillary (V2, red) and mandibular (V1, purple) branches are highlighted with the trigeminal root entry zone outlined (TREZ, blue dashed box). (c) Stitched 100× magnification photomicrograph of MAOB (orange), DAPI (blue) and GFAP (green) of the trigeminal ganglion and representative photomicrographs of Naïve, Sham and ION‐CCI V2 trigeminal ganglion at 400× magnification. (d) Representative photomicrographs within the trigeminal nerve junction of MAOB (orange), DAPI (blue) and glial fibrillary acidic protein immunoreactivity (GFAP) (green) in Naïve, Sham and ION‐CCI rats shown at 400× magnification. Scalebars represent 50 μm, 500 μm or 8 mm.

Qualitative observations of MAO‐B expression in the trigeminal ganglion revealed MAO‐B‐IR in the maxillary division of rats in all groups (Figure 8), this labeling was most prominent in ION‐CCI rats particularly in two distinct locations: firstly surrounding individual sensory neurons, similar in location to satellite glial cells, and secondly running parallel to nerve fibers and capillaries running through the clusters of sensory neurons (Figure 8d). In the TREZ, MAO‐B‐IR was again present in all groups; however, it was much more prominent at the hard boundary between the central and peripheral components of the trigeminal nerve (Figure 8c).

### 3.5 MAO‐B vs. GFAP Co‐Localisation Analysis

**FIGURE 9:**
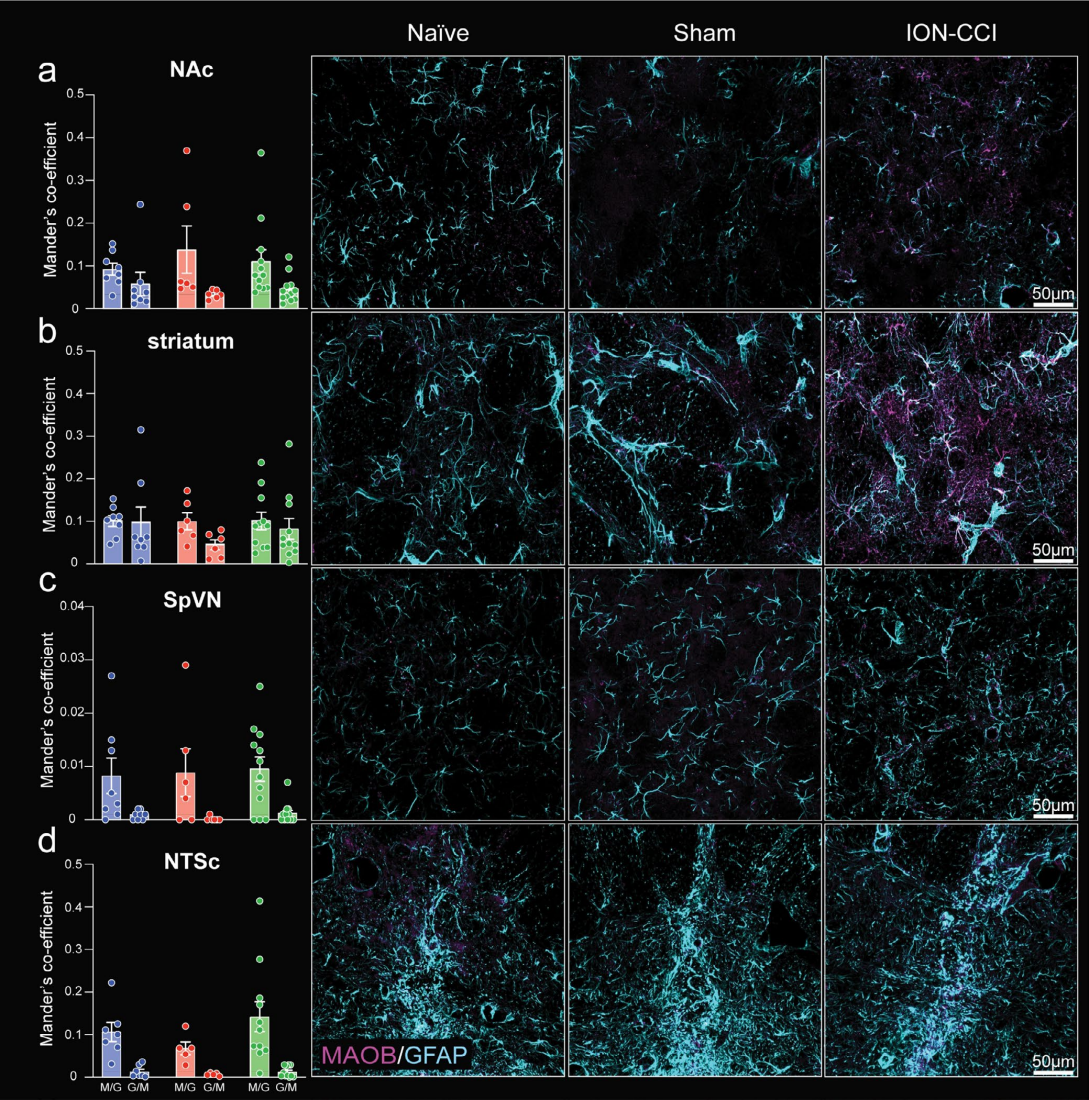
Co‐localisation of GFAP and Monoamine oxidase‐B immunoreactivity. The degree of co‐localisation of GFAP and MAOB immunoreactivity is quantified using Mander's overlap co‐efficient. The co‐efficient are defined as (1) MAO‐B‐IR/GFAP‐IR (M/G), the ratio of the “summed intensities of pixels from the MAO‐B‐IR image for which the intensity in the GFAP‐IR channel is above zero” to the “total intensity in the MAO‐B‐IR channel”; and (2) GFAP‐IR/MAOB‐IR (G/M) is defined as the converse. These data are shown in histograms (left) for (a) Nucleus accumbens shell (NAc), (b) Striatum, (c) Spinal trigeminal nucleus (SpVN), and (d) Commissural subnucleus of the nucleus of the solitary tract (NTSc). Each histogram is shown with representative z‐stack images captured at 400× magnification to visualize co‐localisation of monoamine oxidase B immunoreactive (MAOB‐IR, magenta) and glial fibrillary acidic protein immunoreactive (GFAP‐IR, turquoise) astrocytes in Naïve, Sham and ION‐CCI rats (right). Scale bars represent 50 μm.

**FIGURE 10:**
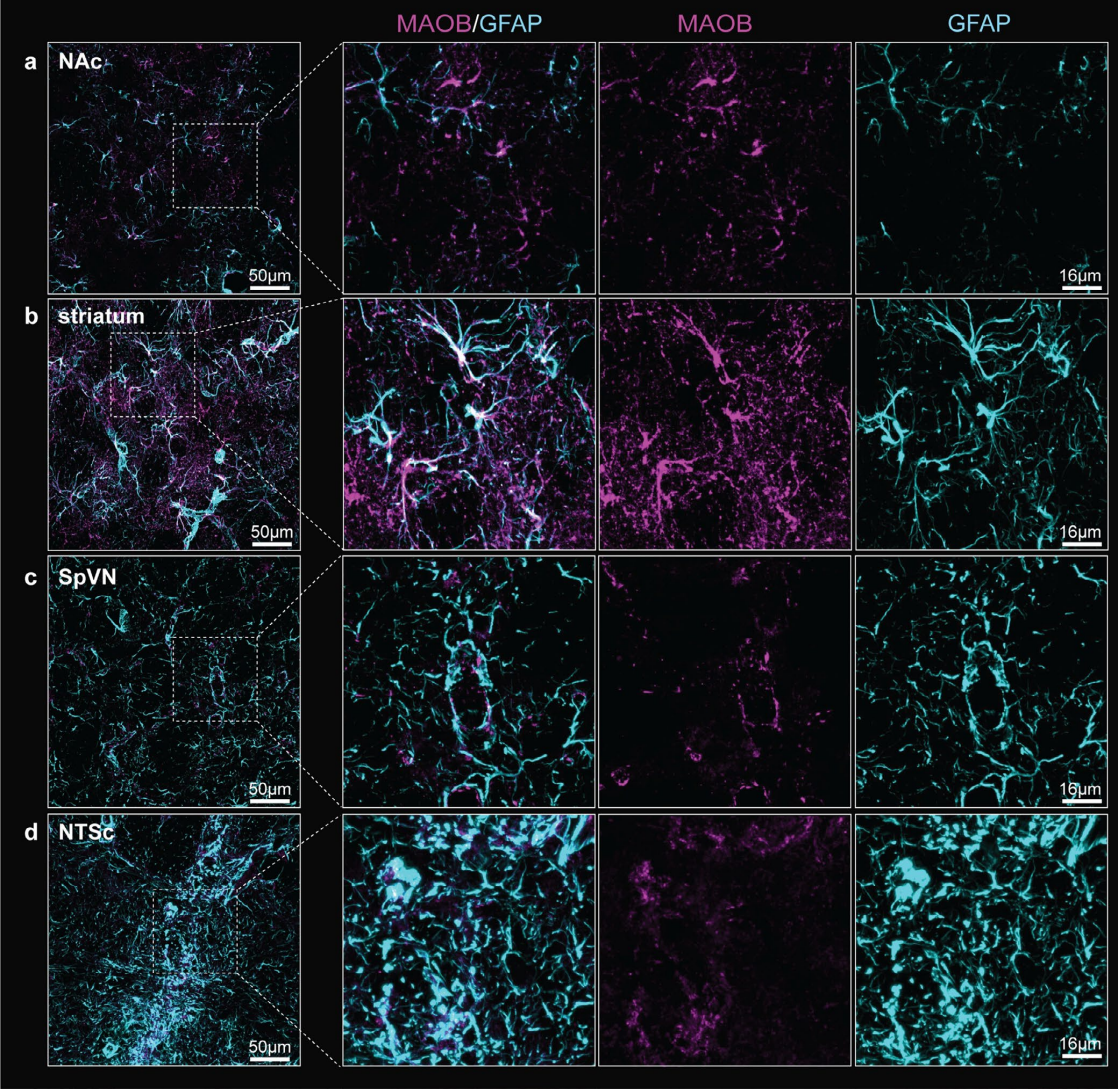
Representative GFAP and Monoamine oxidase‐B Co‐localisation immunoreactivity. Left column shows representative 400× z‐stack micrographs of the ION‐CCI group of rats in the (a) Nucleus accumbens shell (NAc), (b) Striatum, (c) Spinal trigeminal nucleus (SpVN), and (d) Commissural subnucleus of the nucleus of the solitary tract (NTSc). The next column shows the co‐localisation of monoamine oxidase B immunoreactive (MAOB‐IR, magenta) and glial fibrillary acidic protein immunoreactive (GFAP‐IR, turquoise) profiles. The third column shows MAOB‐IR (magenta) alone and the fourth column shows GFAP‐IR (turquoise) alone. Scale bars in the left column represent 50 μm. Scale bars in all other columns represent 16 μm and the images are of 1200× magnification of the region identified in the white dashed boxes in the NAc, striatum, SpVN and NTSc.

In the NAc, striatum, SpVN and NTSc in which there were increased densities of MAO‐B‐IR, with medium to large effect sizes, the co‐localisation with GFAP‐IR was evaluated (Figure 9 and Table S6). In the NAc, each of the three groups showed MAO‐B‐IR, and staining in the ION‐CCI group showed a distinctive “patch‐like” distribution, the NAc also contained GFAP‐IR cells however, only a small proportion of MAO‐B‐IR co‐localized with GFAP‐IR (Figures 9a and 10a: Manders Co‐efficient: MAO‐B‐IR/GFAP‐IR = 0.11 ± 0.03; GFAP‐IR/MAO‐B‐IR = 0.05 ± 0.01). In the lateral aspect of the dorsal striatum, once again the three groups showed MAO‐B‐IR, and staining in the ION‐CCI group showed a similar distinctive “patch‐like” distribution. This region also GFAP‐IR cells once again, only a small proportion of the MAO‐B‐IR co‐localized with GFAP‐IR (Figures 9b and 10b: Manders Co‐efficient: MAO‐B‐IR/GFAP‐IR = 0.1 ± 0.02; GFAP‐IR/MAO‐B‐IR = 0.08 ± 0.02). In the brainstem, the SpVN had only sparse MAO‐B‐IR labeling in Sham rats and much less in Naïve rats whereas there was strong MAO‐B‐IR in ION‐CCI rats. In the SpVN MAO‐B‐IR showed minimal co‐localization with GFAP‐IR (Figures 9c and 10c: Manders Co‐efficient: MAO‐B‐IR/GFAP‐IR = 0.01 ± 0.002; GFAP‐IR/MAO‐B‐IR = 0.001 ± 0.0006). Similarly, in the NTSc, MAO‐B‐IR had minimal co‐localization with GFAP‐IR (Figures 9d and 10d: Manders Co‐efficient: MAO‐B‐IR/GFAP‐IR = 0.14 ± 0.04; GFAP‐IR/MAO‐B‐IR = 0.01 ± 0.004). This lack of colocalization characterized the majority of the MAO‐B‐IR in each of the remaining PET‐SUVr identified regions of interest and includes the infralimbic cortex, the ventral orbital cortex, the piriform cortex, the lateral septal nuclei, and the ventral posterior thalamus (Table S6). High‐magnification split channel images of the colocalization between MAOB‐IR and GFAP‐IR in the NAc, dorsal striatum, SpVN and NTSc of the ION‐CCI group are shown in Figure 10.

## 4 Discussion

We hypothesized that ION‐CCI would evoke increases in [^18^F]‐SMBT‐1 binding and MAO‐B expression in infraorbital nerve recipient regions of the brainstem, specifically in the SpVN, as well as in SpVN recipient supra‐medullary brain regions. We also hypothesized temporal differences in [^18^F]‐SMBT‐1 binding and MAO‐B expression, with an “early” brainstem and “later” forebrain pattern of expression. In the brain, MAO‐B is primarily located on the outer mitochondrial membrane of protoplasmic, perivascular and fibrillary astrocytes, and on the soma of serotonergic neurons of the raphe nucleus (Konradi et al. 1989, Saura et al. 1996, Jaisa‐Aad et al. 2024). Our data reveal longitudinal changes in [^18^F]‐SMBT‐1 binding and MAO‐B expression that we believe reflect increased astrocyte reactivity in rats following ION‐CCI and the development of chronic orofacial neuropathic pain.

Injury‐specific regional changes in astrocyte reactivity occurred with an early post‐injury onset (days 2–7) in the contralateral PAG, the ipsilateral posterior cingulate cortex, the ipsilateral hippocampus and the contralateral entorhinal cortex. This distinct combination of cortical and midbrain structures pattern did not support our initial hypothesis of an early brainstem response and revealed a novel set of brain regions with an early response to trigeminal nerve injury. We also noted a surgery‐specific effect in the ipsilateral medial vestibular nucleus. [^18^F]‐SMBT‐1 binding returned to baseline levels in these regions within 1 week following injury. Late onset, injury‐specific regional changes in astrocyte reactivity (days 14–28) occurred in the trigeminal ganglion, the ipsilateral SpVN, the contralateral dorsolateral striatum and the ipsilateral NAc. We also noted surgery‐specific effects in the ipsilateral trigeminal root entry zone. Two days after the final PET scan (day 30), post‐mortem immunohistochemical analysis of MAO‐B and GFAP immunoreactivity suggested that the increases in SMBT‐1 binding occurred predominantly in GFAP‐negative astrocytes. This is the first longitudinal study identifying changes in astrocyte reactivity following trigeminal nerve injury, and specifically ION‐CCI.

### 4.1 Integrating Longitudinal and Cross‐Sectional Evidence

This is the first longitudinal study recording astrocyte reactivity following trigeminal nerve injury, the time points at which individual rats were evaluated correspond broadly to timepoints in cross‐sectional studies that have addressed this question. While there are no cross‐sectional studies that we are aware of that use MAO‐B expression to define astrocyte reactivity following trigeminal nerve injuries, several studies have used either GFAP or aldehyde dehydrogenase‐1 family member L1 (ALDH1L1) expression for this purpose. Trigeminal nerve injuries in the rat produce marked increases in GFAP in brainstem regions that process nociceptive signals. Following transection, ligation, or constriction of branches of the trigeminal nerve, GFAP expression is reported to increase in the SpVN, the medullary dorsal horn, as well as in the trigeminal ganglion. Evidence that changes in GFAP expression are related directly to trigeminal nerve input was shown by Wang et al. (2010), who showed GFAP increases between 30 and 60 min in the SpVN as a result of direct masseter nerve stimulation, as well as following masseter muscle inflammation with Complete Freunds adjuvant (Guo et al. 2007; Wang et al. 2010).

In rats, transection of the inferior alveolar nerve triggers upregulation of GFAP in the SpVN, between 7 and 14 days after injury (Piao et al. 2006; Okada‐Ogawa et al. 2009). Partial transection of the ION also results in increased GFAP expression in the SpVN (Hu et al. 2024) and ligation or constriction of the nerve (ION‐CCI) significantly increases GFAP in the trigeminal ganglion between 2 and 7 days post‐injury (Donegan et al. 2013; Yoon et al. 2018). As stated, a longitudinal approach to describe the temporal changes in ION‐CCI evoked GFAP expression has not been adopted; however, summarising data from cross‐sectional studies reveals that in the brainstem, GFAP mRNA is upregulated in the SpVN between 1 and 7 days post‐injury (Latrémolière et al. 2008). Increases in GFAP protein determined immunohistochemically are reported in both the SpVN and the medullary dorsal horn (MDH). These increases have been observed 3 days post‐ION‐CCI (SpVN) (Asano et al. 2023); 7 days post‐ION‐CCI (SpVN/MDH) (Asano et al. 2020; Michot et al. 2017; Mah et al. 2017); 14 days post‐ION‐CCI (SpVN/MDH) (Kubíčková et al. 2020); (Dauvergne et al. 2014); 21 days post‐ION‐CCI (SpVN/MDH) (Dieb and Hafidi 2013; Vivanco‐Estela et al. 2025); and 24 days post‐ION‐CCI (SpVN) (Martins et al. 2020). Evidence for supra‐medullary changes in GFAP expression in rats is very limited and is derived from other models of trigeminal pain, including inflammation of the TMJ, manipulations of dental occlusion, and head trauma, and includes increased GFAP expression in the parabrachial nucleus (24 h post‐injury); central nucleus of the amygdala, dorsal hippocampus (3–10 days post‐injury); ventrolateral PAG (10 days post‐injury) (Nascimento et al. 2023; Chen et al. 2007). In all these studies, it is claimed that increased GFAP expression closely correlates with behavioural evidence of hypersensitivity; further, in several of the studies, it is shown that inhibition of astrocyte activation reduces both GFAP expression and pain behaviors. Our data strongly suggest that SMBT‐1 identifies a population of MAO‐B expressing reactive astrocytes that do not colocalise with GFAP. These astrocytes are selectively activated in the early phase of injury in a discrete set of brain regions, including the posterior cingulate cortex, the PAG, the hippocampus, the entorhinal cortex, and the medial vestibular nucleus. A late phase of astrocyte reactivity was observed in the ventral orbital cortex, the infralimbic cortex, the piriform cortex, the NAc, the dorsal striatum, the lateral septal nucleus, the VP thalamus, the SpVN, and the NTSc. Post‐mortem, MAO‐B immunohistochemistry confirmed these increases in the dorsal striatum, the NAc, the SpVN, and the NTSc. Our SMBT1 and MAO‐B data provide evidence of increased astrocyte reactivity in pain‐related brainstem and supra‐medullary sites 28–30 days after ION‐CCI. Our data suggest that MAO‐B and GFAP may reveal different populations of astrocytes with different response profiles to nerve injury.

### 4.2 Functional Significance of “Astrocyte Reactivity”

Glial reactivity identified by increased GFAP expression has been shown to correlate with increased expression of inflammatory mediators such as interleukin‐1‐β, phosphorylated‐ERK, and interferon‐𝛄, along with signs of central sensitization. Reactive GFAP‐IR astrocytes are also known to alter blood–brain barrier permeability, change local calcium dynamics and disrupt GABA‐ergic function, each of which may contribute to the expression of chronic pain (Lee et al. 2022; Matyash and Kettenmann 2010), whether MAOB‐IR reactive astrocytes respond in a similar way remains to be determined. Astrocytes modulate transmission at glutamatergic synapses by both removing extracellular glutamate, and releasing gliotransmitters including glutamate, ATP, and GABA (Vandenberg and Ryan 2013; Sahlender et al. 2014). Reactive astrocytes exhibit calcium waves that can oscillate with infra‐slow frequencies (< 0.1 Hz), it has been shown that these calcium waves can propagate between adjacent astrocytes via gap junctions. Such a mechanism evokes large and long‐lasting NMDA‐mediated currents in thalamocortical neurons via rhythmic gliotransmitter release (Crunelli et al. 2002; Hughes et al. 2011). In abnormal situations, more astrocytes may be recruited resulting in enhanced calcium wave synchrony, increases in amplitude, and amplification of NMDA‐receptor function (Parri and Crunelli 2001; Halassa et al. 2007). Such increases in rhythmic gliotransmitter release and resulting neuronal firing may underlie the infra‐slow oscillations reported in fMRI studies of trigeminal pain pathways in individuals with established chronic orofacial neuropathic pain (Alshelh et al. 2016). We have identified a population of reactive astrocytes in the SpVN 28–30 days after nerve injury based on increased SMBT1 binding and MAOB‐IR. These astrocytes may play a critical role in driving persistent increased neuronal activity in trigeminal pain pathways, via similar mechanisms to those identified above, and thus leading to the establishment of a chronic pain state.

### 4.3 Interpreting Early vs. Late Activation

GFAP‐IR reactive astrocytes have been reported in the PAG following sciatic nerve constriction injury, but to our knowledge not following trigeminal nerve injury. GFAP mRNA was upregulated in the PAG 6 days post spare nerve injury (SNI) and GFAP‐IR was increased in the PAG 6 days and 21 days post‐sciatic nerve CCI (Norman et al. 2010; Mor et al. 2010; Dubový et al. 2018). We show SMBT‐1 binding in the lateral PAG following ION‐CCI 2–7 days post‐injury; this binding is transient and precedes pain chronification. The PAG is a key pain modulatory centre of the brainstem, mediating both facilitatory and inhibitory effects on ascending noxious inputs via a relay in the rostral ventromedial medulla (Chen and Heinricher 2019). The lPAG has a somatotopic organization and the SMBT‐1 binding was located rostrally in the region receiving direct input from the contralateral SpVN (Wiberg et al. 1987). This somatotopic organization is present in humans as shown recently using fMRI; the rostral lPAG is activated by noxious stimulation of the face and the caudal lPAG is activated by noxious stimulation of the body (Tinoco mendoza et al. 2023). The regional specificity of increased SMBT‐1 binding in the trigeminal recipient region of the lPAG contralateral to the injury is consistent with increased astrocyte reactivity driven by neural inputs from the SpVN. Nerve injury evokes both excessive, ongoing excitation in primary afferent pathways (Khan et al. 2002; Chaplan et al. 2003; Amir et al. 2005) as well as decreased postsynaptic inhibition at the primary afferent synapse (Moore et al. 2002). This transient pattern of astrocyte activation may contribute to the acute behavioral responses to nerve injury, including the diminished defensive/active coping behaviors to orofacial stimuli on the injured side (Kang et al. 2024).

We are not aware of any data on the expression of GFAP‐IR reactive astrocytes in the hippocampus for any rat nerve injury models during the early phase (2–7 days post‐injury). However, there are data showing that by day 14 post‐sciatic nerve CCI, that GFAP expression is lower in the ventral hippocampus and dentate gyrus of the hippocampus of injured compared to uninjured rats (Fiore and Austin 2018; Manzhulo et al. 2021). Our data are the first demonstration of MAOB containing reactive astrocytes in the hippocampus 2–7 days post‐ION‐CCI. Increased astrocyte reactivity (i.e., GFAP‐IR) in the hippocampus has been suggested to underly both affective and cognitive deficits observed in neuropathic pain (Fiore and Austin 2018), the exact role of the early onset MAO‐B expression in hippocampus is yet to be investigated. MAO‐B is known to be a key contributor to astrocyte‐derived GABA synthesis. In reactive astrocytes, pathological upregulation of MAO‐B enzymatic activity facilitates aberrant GABA production via the putrescine degradation pathway, resulting in enhanced tonic inhibition that perturbs neuronal membrane potential and impairs synaptic transmission. Inhibition of hippocampal function would lead to impaired affective and cognitive/memory function (Naffaa 2025). In addition, MAO‐B‐catalyzed oxidative deamination generates reactive oxygen species (ROS), such as hydrogen peroxide, amplifying oxidative stress and promoting local neuroinflammation.

We report late onset (14–28 days post‐ION‐CCI), increases in SMBT‐1 binding in the trigeminal ganglion and the trigeminal nerve root entry zone in the PET studies which was confirmed using MAO‐B immunohistochemistry. We showed expression of MAO‐B around ganglion cells, potentially in satellite glial cells, as well as between axons and capillaries traversing the ganglion. MAO‐B was also observed running through ganglion cell clusters and onwards into the region of trigeminal nerve entry into the pons. In addition to the peripheral components of the trigeminal pain pathway, altered SMBT‐1 binding was observed in the ventral orbital cortex, infralimbic cortex, the piriform cortex, NAc, dorsal striatum, lateral septal nuclei, VP thalamus, SpVN and the NTSc.

A recent study described increased GFAP expression in the ventrolateral orbital cortex of female rats, 21 days following ION‐CCI. This study also reported that the optogenetic inhibition of these astrocytes alleviated sensory hypersensitivity, anxiety‐like behaviors and markers of neuroinflammation (Islam et al. 2025). In the infralimbic cortex, NAc and thalamus it is reported that GFAP is not upregulated at 10 days following spared SNI, however the glial excitatory amino acid transporters GLT‐1 and GLAST are significantly upregulated in all of these sites, from this study it is not possible to determine whether this upregulation is within the GFAP‐IR cell population or in an another population of astrocytes (Marcello et al. 2013). Following sciatic nerve CCI, GFAP is upregulated in the shell of the NAc, 28 days after injury (Cazuza et al. 2025). In mice, SNI did not result in changes in GFAP in the NAc 14–21 days after injury (Michailidis et al. 2021), whereas in a mouse model of migraine, 10 days after initiating the head pain, GFAP was upregulated in the NAc (Cropper et al. 2024). In a rat model of oxaliplatin‐dependent neuropathic pain, a significant increase in astrocytes were reported in the striatum 21 days after the neuropathy was initiated (Di Cesare Mannelli et al. 2013). It is interesting to note that in a mouse model of Parkinson disease, reversal of the astrocyte activation observed in the dorsal striatum reverses the hyperalgesia observed in the early development of the condition (Zhang et al. 2024). In the rat VP thalamus, 28 days after a unilateral L5/L6 spinal nerve ligation (SNL) the area of GFAP‐immunoreactivity was increased in injured rats although the number of cells remained unchanged, suggesting an increased reactivity of the resident population (Blaszczyk et al. 2018). To our knowledge nobody has ever investigated astrocytes in the piriform cortex, the lateral septum or the NTSc during the development of chronic pain.

There are only a few previous studies that highlight astrocyte reactivity identified by either GFAP or glial transporter changes in the areas that we have identified with SMBT‐1 binding, confirmed by MAO‐B immunohistochemistry, post‐ION‐CCI. Brain regions such as the orbital cortex, infralimbic cortex, NAc, and thalamus have each been suggested to play important roles in the development and expression of chronic pain, and astrocyte reactivity at these sites is likely to strongly contribute to this. Notably, however, astrocyte reactivity in the piriform cortex, lateral septum, and NTSc remains unexplored, highlighting important future experimental questions.

There are a number of limitations they should be noted. Firstly, we only used male rats and emerging evidence shows that astrocyte reactivity is sexually dimorphic, with differences in GFAP expression, morphology, receptor profiles, and pharmacological responsiveness that vary by brain region and experimental context. These effects appear most pronounced in higher‐order modulatory circuits (e.g., PAG, locus coeruleus, cerebral cortex) rather than primary sensory relays (Boorman and Keay 2022; Nakamoto et al. 2017; Westlund et al. 2026; Reiss et al. 2021; Vivanco‐Estela et al. 2025). However, no study has directly examined sex differences in astrocyte reactivity within the trigeminal subnucleus caudalis, the key relay for trigeminal nociception, because existing work has used only male animals or not reported sex. As a result, it remains unknown whether well‐characterized astrocyte mechanisms in trigeminal pain (e.g., D‐serine/NMDA, mGluR5–P2X3, NF‐κB, A1 states) operate similarly in females. This represents an important limitation and a priority for future studies. Secondly, the potential effects of different anesthetic agents and repeated exposures should be considered. Anesthesia can have direct effects on the biological mechanisms under investigation, and anesthesia can impact the local dynamics of the radiotracer. For instance, the MAOB binding radiotracers [^11^C]AZD9272 and [^11^C]‐L‐deprenyl‐D2 display a 80%–90% reduction in specific binding under sevoflurane compared with ketamine/xylazine anesthesia (Varnas et al. 2021). Furthermore, across species and models, there is experimental evidence that repeated or prolonged anesthesia, especially in the developing brain, can induce long‐lasting astrocyte activation consistent with astrogliosis (Neudecker et al. 2021; Zhang et al. 2020; Liu et al. 2024). However, effects depend on age, anesthetic, dose, and timing, and some adult exposures may instead reduce GFAP or astroglial processes (Dallasen et al. 2011; Wang et al. 2016). Indeed, with respect to repeated isoflurane anesthesia, long (5–6 h) exposure has been shown to increase reactive astrocytes, whilst shorter exposure (2–4 h) may reduce reactive astrocytes (Neudecker et al. 2021; Peng et al. 2022). For these reasons we exposed all animals to the same anesthetic regimen and compared between groups, although the interactions between anesthetic and model cannot be ruled out. A further limitation is that the reversibility of MAO‐B–associated astrocyte reactivity has not been established. No study has yet shown whether MAO‐B PET signal decreases when pain is pharmacologically or physically suppressed, so its sensitivity to pain relief, and therefore its validity as a functional biomarker, remains to be determined.

Our findings suggest that a temporally specific and regionally discrete increase in astrocyte reactivity is a critical contributor to the development and maintenance of chronic neuropathic pain. Early versus late onset changes in astrocyte reactivity likely contribute to different elements of the pain and associated affective and cognitive impairments, a view supported by the observation that drugs that modulate astrocyte reactivity have little effect on the expression of acute pain (Zhuang et al. 2006; Wang et al. 2009). Targeting specific astrocyte subpopulations may offer more effective treatments for chronic neuropathic pain. Our approach enables longitudinal tracking of astrocyte reactivity in individual animals as pain progresses, allowing evaluation of therapeutic compounds at various stages. The ability to perform SMBT‐1 PET imaging in humans with orofacial neuropathic pain enhances translational potential, helping identify brain regions most likely to yield clinical benefits.

## Author Contributions

Conceptualization: G.M.E., W.N., M.B.H., K.A.K., and L.A.H. Methodology: L.S.C., J.W.M.K., O.I.D., and H.L. Software: L.S.C., O.I.D., B.J., M.L., and G.M.E. Validation: J.W.M.K., H.L., C.L., and B.J. Formal analysis: L.S.C., J.W.M.K., O.I.D., H.L., and G.M.E. Investigation: J.W.M.K., S.S., M.S., C.L., G.M.E., and R.M. Resources: M.L., W.N., M.B.H., and R.M. Data Curation: L.S.C., O.I.D., H.L., S.S., M.S., and C.L. Writing – original draft: L.S.C., J.W.M.K., K.A.K., and L.A.H. Writing – review and editing: All authors. Visualization: L.S.C., J.W.M.K., O.I.D., and H.L. Supervision: K.A.K., and L.A.H. Project administration: L.A.H., and K.A.K. Funding acquisition: L.A.H., and K.A.K.

## Funding

This work was supported by the National Health and Medical Research Council (grant no. 1130280).

## Conflicts of Interest

The authors declare no conflicts of interest.

## Supporting information


**Data S1:** PET and injury‐related changes in autonomic and physiological activity.

**Figure S1:** Time activity curves (TAC) of [^18^F]‐SMBT‐1 decay over time. Each animal Naïve control (*n* = 8), Sham (*n* = 6) or (ION‐CCI) (*n* = 11) group was entered into the positron emission tomography (PET) scanner for a period of 60‐min following bolus injection with [^18^F]‐SMBT‐1. A total of 16 frames were collected corresponding with set time points from injection, with the standardized uptake value ratio, normalized to the whole brain, extracted from each frame to determine the overall decay constant of SMBT‐1 within each group at each time point relative to injury. Five lines corresponding to the five PET sessions: ‐day 7 (dark blue), day 2 (orange), day 7 (light green), day 14 (light blue), and day 28 (pink) were plotted within animals from (a) Naïve, (b) Sham, or (c) ION‐CCI. (d) **Example slices of the whole brain mask used for generating SUVr values in each experimental cohort**. Nonlinear affine co‐registration was performed to warp each animal's anatomical (CT) data to the SIGMA preclinical brain template. These values were stored and applied to the corresponding PET image data of each animal, before extracting a global mean value of [^18^F]‐SMBT‐1 SUV within every brain voxel of the template image to be used for calculation of wholebrain SUVr image sets.

**Figure S2:** Percentage standardized uptake value ratio relative to group baseline in early and late change [^18^F]‐SMBT1 regions. (a) Regions displaying a significant effect of time in ION‐CCI animals such that the greatest change occurred at early (days +2 and +7) relative to late time points following injury. (b) Extracted SUVr values normalized to pre‐injury baseline in naïve (blue points and lines; *n* = 8), sham (red points and lines; *n* = 6), and ION‐CCI (green points and lines; *n* = 11). Note that the largest percentage changes in SUVr are observed in ION‐CCI animals, and specifically at day +2 following injury. (c) Regions displaying a significant effect of time in ION‐CCI animals such that the greatest change occurred at late (days +14 and +28) relative to late time points following injury. (d) Extracted SUVr values normalized to pre‐injury baseline in naïve (blue points and lines; *n* = 8), sham (red points and lines; *n* = 6), and ION‐CCI (green points and lines; *n* = 11). Note that the largest percentage change in SUVr are observed in ION‐CCI animals, and specifically at day +28 following injury. *Indicates significance between ION‐CCI and Naïve cohorts, and # between ION‐CCI and Sham cohorts determined through post hoc two‐sample *t*‐tests. Binding increases are indicated by the hot color scale overlaid onto a T2‐weighted anatomical template. Slice locations relative to Bregma are indicated at the top right of each coronal slice. contra = contralateral.

**Table S1:** Location from bregma (in millimeters), cluster size (Ke), *t*‐value, and standardized uptake value ratio (SUVr) normalized to a value of 1 relative to day −7 extracted from clusters that display altered radioligand binding over time in infraorbital nerve chronic constriction injury animals (ION‐CCI; *n* = 11). ipsi = ipsilateral; contra = contralateral; SpVN = spinal trigeminal nucleus; Cb = cerebellar; Trig. = trigeminal; TREZ = trigeminal root entry zone; MGN = medial geniculate nucleus; NTSc = Nucleus of the solitary tract, commissural region; VPL = ventral posterolateral thalamus; M1 = primary motor cortex; PAG = midbrain periaqueductal gray matter; PCC = posterior cingulate cortex.

**Table S2:** Naïve (*n* = 8) rats standardized uptake value ratio (SUVr) values normalized to a value of 1 relative to day −7 extracted from clusters that display altered SMBT‐1 radioligand binding over time in ION‐CCI rats. ipsi = ipsilateral; contra = contralateral; SpVN = spinal trigeminal nucleus; Cb = cerebellar; trig. = trigeminal; TREZ = trigeminal root entry zone; MGN = medial geniculate nucleus; VPL = ventral posterolateral thalamus; M1 = primary motor cortex; NTSc = Nucleus of the solitary tract, commissural region; PAG = midbrain periaqueductal gray matter; PCC = posterior cingulate cortex.

**Table S3:** Sham (*n* = 6) rats standardized uptake value ratio (SUVr) values normalized to a value of 1 relative to day −7 extracted from clusters that display altered SMBT‐1 radioligand binding over time in ION‐CCI rats. ipsi = ipsilateral; contra = contralateral; SpVN = spinal trigeminal nucleus; Cb = cerebellar; trig. = trigeminal; TREZ = trigeminal root entry zone; MGN = medial geniculate nucleus; NTSc = Nucleus of the solitary tract, commissural region; VPL = ventral posterolateral thalamus; M1 = primary motor cortex; PAG = midbrain periaqueductal gray matter; PCC = posterior cingulate cortex.

**Table S4:** The integrated density of Monoamine oxidase‐B immunoreactivity in Naïve, Sham or ION‐CCI rats are presented with standard error of the mean (SEM). *Indicates moderate effect sizes compared to Naïve and #indicated moderate effect size compared to Naïve and, ^#^indicates moderate effect size compared to Sham (0.5 < d > 0.8). NAc nucleus accumbens; VP = ventral posterior thalamus; SpVN = spinal trigeminal nucleus; NTSc = commissural nucleus of the nucleus tractus solitarius.

**Table S5:** Effect sizes and null hypothesis statistical testing. Effect sizes (Cohen's d) and their 95% confidence intervals were calculated using bias‐corrected and accelerated bootstrap resampling (5000 bootstrap samples per test) using the web application built by Hung Nguyen (estimationstats.com), which uses the Python code developed by Ho et al. [85]. *Indicates moderate effect sizes (0.5 < *d* > 0.8).

**Table S6:** The degree of co‐localisation of GFAP and MAOB immunoreactivity tested using Mander's overlap co‐efficient. The co‐efficient were defined as (1) MAO‐B‐IR/GFAP‐IR (M/G), the ratio of the “summed intensities of pixels from the MAO‐B‐IR image for which the intensity in the GFAP‐IR channel is above zero” to the “total intensity in the MAO‐B‐IR channel”; and (2) GFAP‐IR/MAOB‐IR (G/M) is defined as the converse. NAc = nucleus accumbens; SpVN = spinal trigeminal nucleus; NTSc = commissural subnucleus of the nucleus of the solitary tract.

**Table S7:** Body weight change throughout experimental timeline, physiological changes, and anesthetic monitoring within the positron emission tomography scanner at each experimental time ION‐CCI (*n* = 11), Sham (*n* = 6), and Naïve (*n* = 8) groups.

**Table S8:** [^18^F]‐SMBT‐1 time activity curve values within candidate reference regions: Cerebellum, left and right thalamus, and infralimbic cortices. Each value represents the mean ± SEM standardized SMBT‐1 uptake value (SUV) within these sites in each experimental cohort: Naïve, Sham or ION‐CCI rats, averaged across all five experimental time points.

**Table S9:** Average initial uptake and decay of [^18^F]‐SMBT‐1 whole brain SUV within ION‐CCI, Naïve, and Sham cohorts. Single factor ANOVA was conducted to assess between group variance, with coefficient of variation testing performed to assess within‐group variance of initial tracer uptake.

## Data Availability

The data that support the findings of this study are available from the corresponding author upon reasonable request.
