# Supplementary figures and images for: Longitudinal Tracking of Astrocyte Reactivity During the Development of Chronic Orofacial Neuropathic Pain Using [ 18F]‐SMBT‐1 Positron‐Emission Tomography
Source: Glia. 2026 Jun 18;74(8):e70182. doi: 10.1002/glia.70182 (PMC13278361; doi:10.1002/glia.70182)

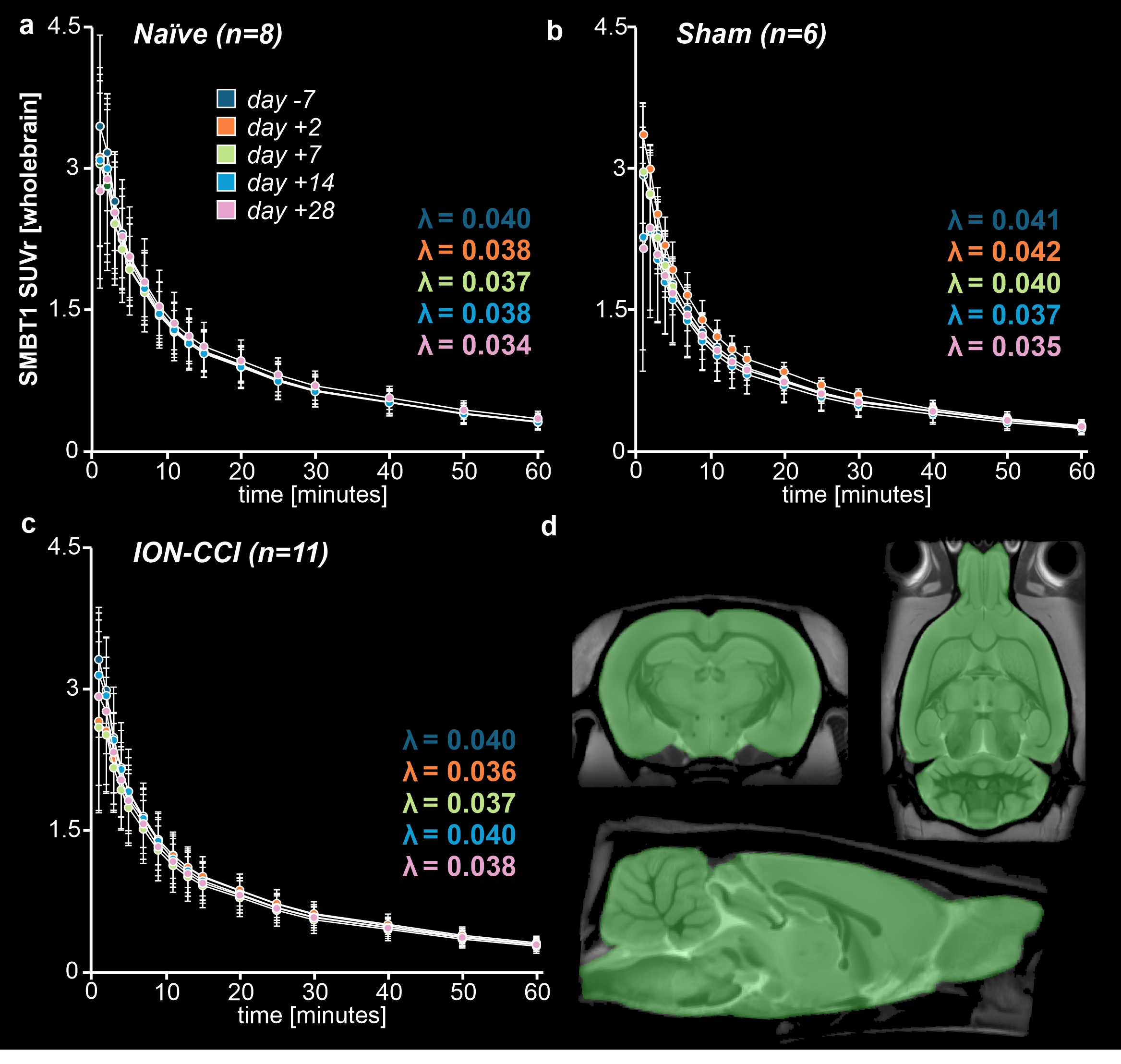

Supplement: Supplementary file 2 — Figure S1: Time activity curves (TAC) of [18F]‐SMBT‐1 decay over time. Each animal Naïve control (n = 8), Sham (n = 6) or (ION‐CCI) (n = 11) group was entered into the positron emission tomography (PET) scanner for a period of 60‐min following bolus injection with [18F]‐SMBT‐1. A total of 16 frames were collected corresponding with set time points from injection, with the standardized uptake value ratio, normalized to the whole brain, extracted from each frame to determine the overall decay constant of SMBT‐1 within each group at each time point relative to injury. Five lines corresponding to the five PET sessions: ‐day 7 (dark blue), day 2 (orange), day 7 (light green), day 14 (light blue), and day 28 (pink) were plotted within animals from (a) Naïve, (b) Sham, or (c) ION‐CCI. (d) Example slices of the whole brain mask used for generating SUVr values in each experimental cohort. Nonlinear affine co‐registration was performed to warp each animal's anatomical (CT) data to the SIGMA preclinical brain template. These values were stored and applied to the corresponding PET image data of each animal, before extracting a global mean value of [18F]‐SMBT‐1 SUV within every brain voxel of the template image to be used for calculation of wholebrain SUVr image sets. [file GLIA-74-0-s005.jpg]

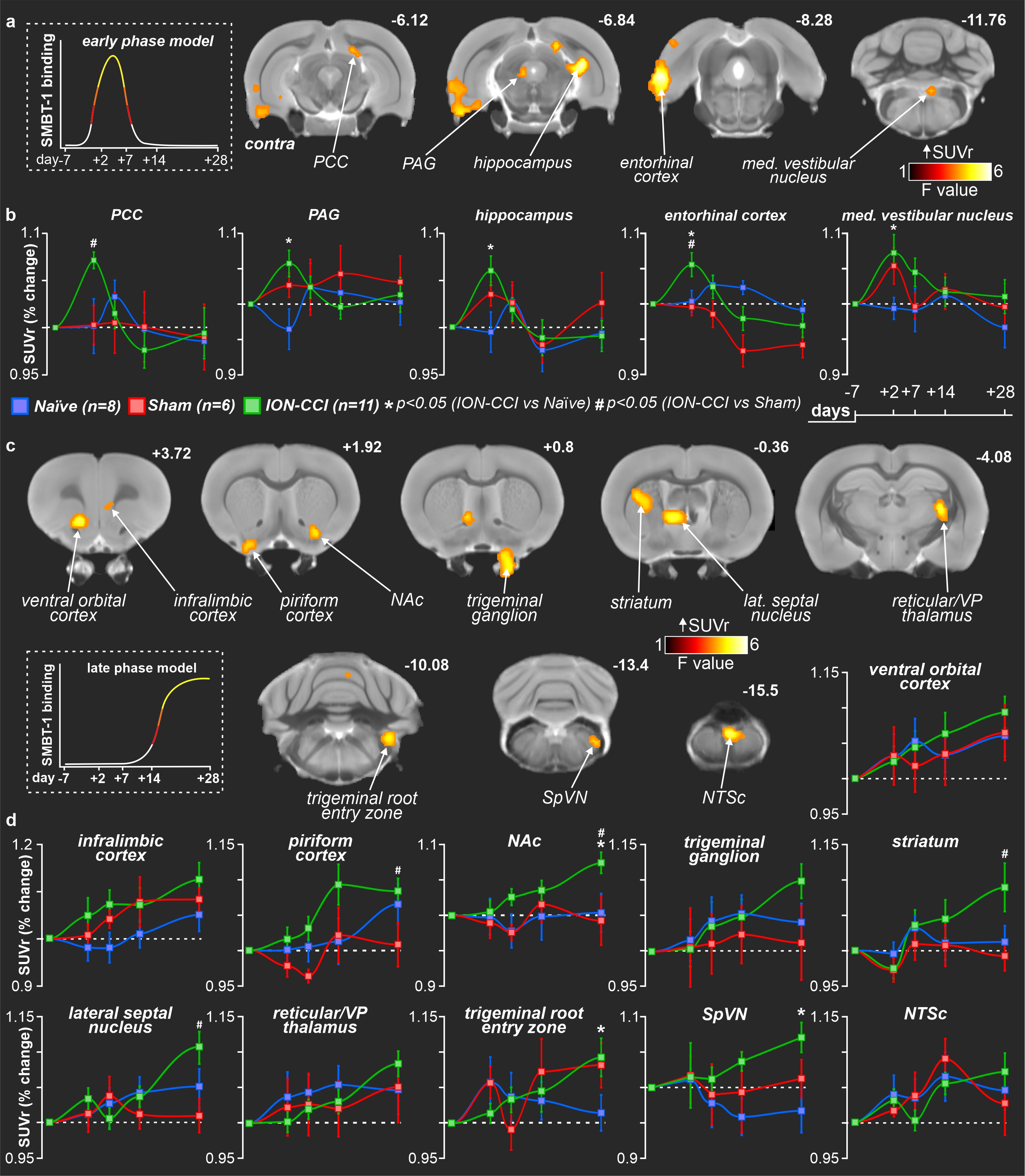

Supplement: Supplementary file 3 — Figure S2: Percentage standardized uptake value ratio relative to group baseline in early and late change [18F]‐SMBT1 regions. (a) Regions displaying a significant effect of time in ION‐CCI animals such that the greatest change occurred at early (days +2 and +7) relative to late time points following injury. (b) Extracted SUVr values normalized to pre‐injury baseline in naïve (blue points and lines; n = 8), sham (red points and lines; n = 6), and ION‐CCI (green points and lines; n = 11). Note that the largest percentage changes in SUVr are observed in ION‐CCI animals, and specifically at day +2 following injury. (c) Regions displaying a significant effect of time in ION‐CCI animals such that the greatest change occurred at late (days +14 and +28) relative to late time points following injury. (d) Extracted SUVr values normalized to pre‐injury baseline in naïve (blue points and lines; n = 8), sham (red points and lines; n = 6), and ION‐CCI (green points and lines; n = 11). Note that the largest percentage change in SUVr are observed in ION‐CCI animals, and specifically at day +28 following injury. *Indicates significance between ION‐CCI and Naïve cohorts, and # between ION‐CCI and Sham cohorts determined through post hoc two‐sample t‐tests. Binding increases are indicated by the hot color scale overlaid onto a T2‐weighted anatomical template. Slice locations relative to Bregma are indicated at the top right of each coronal slice. contra = contralateral. [file GLIA-74-0-s007.jpg]
