# Supplementary material for: Longitudinal Tracking of Astrocyte Reactivity During the Development of Chronic Orofacial Neuropathic Pain Using [ 18F]‐SMBT‐1 Positron‐Emission Tomography
Source: Glia. 2026 Jun 18;74(8):e70182. doi: 10.1002/glia.70182 (PMC13278361; doi:10.1002/glia.70182)
Supplement: Supplementary file 4 — Table S1: Location from bregma (in millimeters), cluster size (Ke), t‐value, and standardized uptake value ratio (SUVr) normalized to a value of 1 relative to day −7 extracted from clusters that display altered radioligand binding over time in infraorbital nerve chronic constriction injury animals (ION‐CCI; n = 11). ipsi = ipsilateral; contra = contralateral; SpVN = spinal trigeminal nucleus; Cb = cerebellar; Trig. = trigeminal; TREZ = trigeminal root entry zone; MGN = medial geniculate nucleus; NTSc = Nucleus of the solitary tract, commissural region; VPL = ventral posterolateral thalamus; M1 = primary motor cortex; PAG = midbrain periaqueductal gray matter; PCC = posterior cingulate cortex. [file GLIA-74-0-s002.docx]

**Supplementary table 1.** Location from bregma (in millimetres), cluster size (Ke), t-value, and standardized uptake value ratio (SUVr) normalized to a value of 1 relative to day -7 extracted from clusters that display altered radioligand binding over time in infraorbital nerve chronic constriction injury animals (ION-CCI; n=11). ipsi = ipsilateral; contra = contralateral; SpVN = spinal trigeminal nucleus; Cb = cerebellar; Trig. = trigeminal; TREZ = trigeminal root entry zone; MGN = medial geniculate nucleus; NTSc = Nucleus of the solitary tract, commissural region; VPL = ventral posterolateral thalamus; M1 = primary motor cortex; PAG = midbrain periaqueductal gray matter; PCC = posterior cingulate cortex.

|  |  |  |  | **ION-CCI** | | | | |
| --- | --- | --- | --- | --- | --- | --- | --- | --- |
| **region** | **bregma** | **Ke** | **t-value** | **day +2 SUVr (±SEM)** | **day +7 SUVr (±SEM)** | **day +14 SUVr (±SEM)** | **day +28 SUVr (±SEM)** | **p-value** |
| ***early onset model*** |  |  |  |  |  |  |  |  |
| medial vestibular nucleus | -13.76 | 39 | 3.55 | 1.07±0.03 | 1.04±0.02 | 1.02±0.02 | 1.01±0.02 | 0.01 |
| contra entorhinal cortex | -7.2 | 504 | 5.35 | 1.06±0.02 | 1.02±0.02 | 0.98±0.02 | 0.97±0.02 | <0.001 |
| contra lateral PAG | -7.56 | 22 | 3.53 | 1.06±0.02 | 1.02±0.01 | 1.00±0.02 | 1.01±0.02 | <0.001 |
| ipsi hippocampus | -7.32 | 142 | 5.32 | 1.06±0.02 | 1.02±0.01 | 0.99±0.02 | 0.99±0.02 | <0.001 |
| ipsi PCC | -7.2 | 35 | 3.75 | 1.05±0.01 | 1.00±0.01 | 0.97±0.01 | 0.99±0.01 | <0.001 |
| ***late onset model*** |  |  |  |  |  |  |  |  |
| NTSc | -15.48 | 68 | 4.90 | 1.03±0.02 | 1.00±0.01 | 1.05±0.01 | 1.07±0.03 | 0.003 |
| ipsi SpVN | -13.36 | 32 | 3.85 | 1.01±0.01 | 1.01±0.02 | 1.04±0.01 | 1.07±0.02 | <0.001 |
| Cb Crus layer 6 | -11.64 | 156 | 4.27 | 1.03±0.02 | 1.02±0.02 | 1.06±0.01 | 1.08±0.01 | <0.001 |
| ipsi TREZ | -9.84 | 53 | 3.98 | 1.01±0.01 | 1.03±0.02 | 1.05±0.03 | 1.09±0.03 | 0.002 |
| ipsi MGN | -5.2 | 62 | 4.05 | 1.00±0.01 | 1.00±0.02 | 1.03±0.02 | 1.07±0.02 | <0.001 |
| ipsi VPL + reticular thalamus | -3.12 | 56 | 4.44 | 1.01±0.01 | 1.03±0.01 | 1.04±0.02 | 1.08±0.02 | <0.001 |
| contra dorsolateral striatum | -0.66 | 87 | 3.87 | 0.97±0.02 | 1.04±0.02 | 1.04±0.03 | 1.09±0.03 | <0.001 |
| contra lateral septal nucleus | 0.00 | 112 | 4.48 | 1.03±0.01 | 1.00±0.01 | 1.04±0.01 | 1.10±0.02 | <0.001 |
| ipsi Trig. Ganglion | 0.84 | 84 | 4.02 | 1.00±0.02 | 1.04±0.03 | 1.05±0.03 | 1.10±0.02 | 0.001 |
| ipsi. lateral accumbens shell | 1.92 | 59 | 3.82 | 1.00±0.02 | 1.03±0.01 | 1.04±0.01 | 1.08±0.01 | <0.001 |
| contra piriform cortex | 2.16 | 101 | 4.81 | 1.02±0.02 | 1.03±0.02 | 1.09±0.03 | 1.08±0.02 | <0.001 |
| ipsi M1 | 2.52 | 24 | 3.57 | 1.02±0.02 | 1.05±0.02 | 1.09±0.03 | 1.09±0.03 | 0.01 |
| contra ventral orbital cortex | 3.72 | 72 | 4.49 | 1.02±0.02 | 1.04±0.02 | 1.06±0.03 | 1.09±0.02 | 0.002 |
| ipsi infralimbic cortex | 4.16 | 22 | 3.00 | 1.02±0.02 | 1.04±0.02 | 1.06±0.03 | 1.09±0.02 | 0.002 |
