# Supplementary material for: Longitudinal Tracking of Astrocyte Reactivity During the Development of Chronic Orofacial Neuropathic Pain Using [ 18F]‐SMBT‐1 Positron‐Emission Tomography
Source: Glia. 2026 Jun 18;74(8):e70182. doi: 10.1002/glia.70182 (PMC13278361; doi:10.1002/glia.70182)
Supplement: Supplementary file 5 — Table S2: Naïve (n = 8) rats standardized uptake value ratio (SUVr) values normalized to a value of 1 relative to day −7 extracted from clusters that display altered SMBT‐1 radioligand binding over time in ION‐CCI rats. ipsi = ipsilateral; contra = contralateral; SpVN = spinal trigeminal nucleus; Cb = cerebellar; trig. = trigeminal; TREZ = trigeminal root entry zone; MGN = medial geniculate nucleus; VPL = ventral posterolateral thalamus; M1 = primary motor cortex; NTSc = Nucleus of the solitary tract, commissural region; PAG = midbrain periaqueductal gray matter; PCC = posterior cingulate cortex. [file GLIA-74-0-s003.docx]

**Supplementary table 2.** Naïve (n=8) rats standardized uptake value ratio (SUVr) values normalized to a value of 1 relative to day -7 extracted from clusters that display altered SMBT-1 radioligand binding over time in ION-CCI rats. ipsi = ipsilateral; contra = contralateral; SpVN = spinal trigeminal nucleus; Cb = cerebellar; trig. = trigeminal; TREZ = trigeminal root entry zone; MGN = medial geniculate nucleus; VPL = ventral posterolateral thalamus; M1 = primary motor cortex; NTSc = Nucleus of the solitary tract, commissural region; PAG = midbrain periaqueductal gray matter; PCC = posterior cingulate cortex.

|  | **Naïve** | | | | |
| --- | --- | --- | --- | --- | --- |
| **region** | **day +2 SUVr (±SEM)** | **day +7 SUVr (±SEM)** | **day +14 SUVr (±SEM)** | **day +28 SUVr (±SEM)** | **p-value** |
| ***early onset model*** |  |  |  |  |  |
| medial vestibular nucleus | 0.99±0.03 | 0.99±0.03 | 1.01±0.03 | 0.97±0.04 | 0.74 |
| contra entorhinal cortex | 1.00±0.02 | 1.02±0.01 | 1.02±0.01 | 0.99±0.01 | 0.22 |
| contra lateral PAG | 0.96±0.03 | 1.03±0.03 | 1.02±0.03 | 1.00±0.03 | 0.67 |
| ipsi hippocampus | 0.99±0.02 | 1.03±0.02 | 0.98±0.02 | 0.99±0.02 | 0.50 |
| ipsi PCC | 1.00±0.02 | 1.03±0.01 | 1.00±0.01 | 0.99±0.01 | 0.53 |
| ***late onset model*** |  |  |  |  |  |
| NTSc | 1.04±0.03 | 1.03±0.03 | 1.06±0.03 | 1.04±0.03 | 0.59 |
| ipsi SpVN | 1.01±0.03 | 0.98±0.03 | 0.96±0.03 | 0.97±0.03 | 0.69 |
| Cb Crus layer 6 | 0.96±0.02 | 0.95±0.02 | 1.02±0.02 | 1.02±0.01 | 0.007 |
| ipsi TREZ | 1.06±0.02 | 1.04±0.03 | 1.03±0.03 | 1.01±0.03 | 0.59 |
| ipsi MGN | 1.01±0.02 | 1.05±0.02 | 1.03±0.02 | 1.03±0.03 | 0.57 |
| ipsi VPL + reticular thalamus | 1.04±0.03 | 1.04±0.02 | 1.05±0.02 | 1.07±0.03 | 0.50 |
| contra dorsolateral striatum | 0.99±0.02 | 1.03±0.02 | 1.01±0.02 | 1.01±0.02 | 0.72 |
| contra lateral septal nucleus | 1.01±0.02 | 1.03±0.02 | 1.04±0.02 | 1.05±0.02 | 0.40 |
| ipsi trig. ganglion | 1.02±0.03 | 1.04±0.03 | 1.05±0.03 | 1.04±0.03 | 0.65 |
| ipsi. lateral accumbens shell | 1.00±0.03 | 0.98±0.02 | 1.00±0.03 | 1.00±0.03 | 0.96 |
| contra piriform cortex | 1.00±0.02 | 1.00±0.02 | 1.01±0.03 | 1.07±0.03 | 0.30 |
| ipsi M1 | 0.99±0.03 | 0.95±0.04 | 1.00±0.03 | 1.00±0.04 | 0.76 |
| contra ventral orbital cortex | 1.03±0.03 | 1.05±0.03 | 1.03±0.02 | 1.06±0.03 | 0.58 |
| ipsi infralimbic cortex | 0.98±0.03 | 0.98±0.03 | 1.01±0.03 | 1.05±0.04 | 0.47 |
