# Supplementary material for: Longitudinal Tracking of Astrocyte Reactivity During the Development of Chronic Orofacial Neuropathic Pain Using [ 18F]‐SMBT‐1 Positron‐Emission Tomography
Source: Glia. 2026 Jun 18;74(8):e70182. doi: 10.1002/glia.70182 (PMC13278361; doi:10.1002/glia.70182)
Supplement: Supplementary file 7 — Table S4: The integrated density of Monoamine oxidase‐B immunoreactivity in Naïve, Sham or ION‐CCI rats are presented with standard error of the mean (SEM). *Indicates moderate effect sizes compared to Naïve and #indicated moderate effect size compared to Naïve and, #indicates moderate effect size compared to Sham (0.5 < d > 0.8). NAc nucleus accumbens; VP = ventral posterior thalamus; SpVN = spinal trigeminal nucleus; NTSc = commissural nucleus of the nucleus tractus solitarius. [file GLIA-74-0-s011.docx]

**Supplementary table 4.** The integrated density of Monoamine oxidase-B immunoreactivity in Naïve, Sham or ION-CCI rats are presented with standard error of the mean (SEM). * Indicates moderate effect sizes compared to Naïve and #indicated moderate effect size compared to Naïve and, ^#^ indicates moderate effect size compared to Sham (0.5 < d > 0.8). NAc nucleus accumbens; VP = ventral posterior thalamus; SpVN = spinal trigeminal nucleus; NTSc = commissural nucleus of the nucleus tractus solitarius.

| region of interest | Naïve | Sham | ION-CCI |
| --- | --- | --- | --- |
| infralimbic cortex | 282018 ± 29149 | **337437 ± 42994*** | 343013 ± 98397 |
| ventral orbital cortex | 132183 ± 32282 | 137923 ± 29255 | 168708 ± 44220 |
| NAc | 145035 ± 9931 | 153701 ± 14942 | **205509 ± 42476*** |
| piriform cortex | 169038 ± 22301 | 163850 ± 27738 | 199701 ± 29389 |
| lateral septal nuclei | 223983 ± 41621 | 200895 ± 53777 | 228379 ± 22935 |
| dorsal striatum | 204188 ± 51583 | 168997 ± 34375 | **283485 ± 79246^#^** |
| VP thalamus | 44941 ± 7033 | 51752 ± 13258 | 49851 ± 7846 |
| SpVN | 43641 ± 7067 | 39773 ± 8105 | **64683 ± 10521*^#^** |
| NTSc | 142708 ± 40111 | 98101 ± 15596 | **120316 ± 30702^#^** |
