# Supplementary material for: Longitudinal Tracking of Astrocyte Reactivity During the Development of Chronic Orofacial Neuropathic Pain Using [ 18F]‐SMBT‐1 Positron‐Emission Tomography
Source: Glia. 2026 Jun 18;74(8):e70182. doi: 10.1002/glia.70182 (PMC13278361; doi:10.1002/glia.70182)
Supplement: Supplementary file 8 — Table S5: Effect sizes and null hypothesis statistical testing. Effect sizes (Cohen's d) and their 95% confidence intervals were calculated using bias‐corrected and accelerated bootstrap resampling (5000 bootstrap samples per test) using the web application built by Hung Nguyen (estimationstats.com), which uses the Python code developed by Ho et al. [85]. *Indicates moderate effect sizes (0.5 < d > 0.8). [file GLIA-74-0-s012.docx]

**Supplementary table 5.** Effect sizes and null hypothesis statistical testing. Effect sizes (Cohen's d) and their 95% confidence intervals were calculated using bias-corrected and accelerated bootstrap resampling (5000 bootstrap samples per test) using the web application built by Hung Nguyen (estimationstats.com), which uses the Python code developed by Ho, et al., [85]. * Indicates moderate effect sizes (0.5 < *d* > 0.8).

| Region of interest | Naïve vs Sham | | Naïve vs ION-CCI | | Sham vs ION-CCI | |
| --- | --- | --- | --- | --- | --- | --- |
|  | Cohen’s  d | t-test  two-sided | Cohen’s  d | t-test  two-sided  (p-value) | Cohen’s  d | t-test  two-sided |
| infralimbic cortex | **0.609* [95.0%CI -0.812, 1.81]** | P=0.291 | 0.219 [95.0%CI -1.08, 0.748] | P=0.874 | 0.0193 [95.0%CI -1.69, 0.632] | P=0.99 |
| ventral orbital cortex | 0.0722 [95.0%CI -1.19, 1.48] | P=0.885 | 0.274 [95.0%CI -0.793, 0.958] | P=0.623 | 0.231 [95.0%CI -0.768, 0.953] | P=0.71 |
| NAc | 0.26 [95.0%CI -0.834, 1.54] | P=0.635 | **0.505* [95.0%CI -0.376, 1.03]** | P=0.337 | 0.419 [95.0%CI -0.516, 0.947] | P=0.474 |
| Piriform  cortex | -0.0821 [95.0%CI -1.52, 1.21] | P=0.886 | 0.344 [95.0%CI -0.538, 1.13] | P=0.49 | 0.387 [95.0%CI -0.789, 1.1] | P=0.463 |
| septum | -0.192 [95.0%CI -1.49, 1.24] | P=0.705 | 0.0481 [95.0%CI -1.11, 1.13] | P=0.918 | 0.278 [95.0%CI -1.41, 1.58] | P=0.589 |
| striatum | -0.304 [95.0%CI -1.36, 0.95] | P=0.626 | 0.354 [95.0%CI -0.714, 1.03] | P=0.488 | **0.52* [95.0%CI -0.283, 1.19]** | P=0.342 |
| thalamus | 0.263 [95.0%CI -1.14, 1.58] | P=0.641 | 0.2 [95.0%CI -0.738, 1.01] | P=0.672 | -0.0657 [95.0%CI -1.38, 0.957] | P=0.888 |
| SpVN | is -0.194 [95.0%CI -1.39, 1.0] | P=0.707 | **0.677* [95.0%CI -0.187, 1.45]** | P=0.156 | **0.774* [95.0%CI -0.105, 1.57]** | P=0.143 |
| NTSc | -0.367 [95.0%CI -1.29, 1.22] | P=0.605 | -0.384 [95.0%CI -1.58, 0.639] | P=0.41 | **0.486* [95.0%CI -0.393, 1.38]** | P=0.338 |
