# Supplementary material for: Longitudinal Tracking of Astrocyte Reactivity During the Development of Chronic Orofacial Neuropathic Pain Using [ 18F]‐SMBT‐1 Positron‐Emission Tomography
Source: Glia. 2026 Jun 18;74(8):e70182. doi: 10.1002/glia.70182 (PMC13278361; doi:10.1002/glia.70182)
Supplement: Supplementary file 9 — Table S6: The degree of co‐localisation of GFAP and MAOB immunoreactivity tested using Mander's overlap co‐efficient. The co‐efficient were defined as (1) MAO‐B‐IR/GFAP‐IR (M/G), the ratio of the “summed intensities of pixels from the MAO‐B‐IR image for which the intensity in the GFAP‐IR channel is above zero” to the “total intensity in the MAO‐B‐IR channel”; and (2) GFAP‐IR/MAOB‐IR (G/M) is defined as the converse. NAc = nucleus accumbens; SpVN = spinal trigeminal nucleus; NTSc = commissural subnucleus of the nucleus of the solitary tract. [file GLIA-74-0-s008.docx]

**Supplementary table 6.** The degree of co-localisation of GFAP and MAOB immunoreactivity tested using Mander’s overlap co-efficient. The co-efficient were defined as 1) MAO-B-IR/GFAP-IR (M/G), the ratio of the "summed intensities of pixels from the MAO-B-IR image for which the intensity in the GFAP-IR channel is above zero" to the "total intensity in the MAO-B-IR channel"; and 2) GFAP-IR/MAOB-IR (G/M) is defined as the converse. NAc = nucleus accumbens; SpVN = spinal trigeminal nucleus; NTSc = commissural subnucleus of the nucleus of the solitary tract.

| Region of interest | Naïve | | Sham | | ION-CCI | |
| --- | --- | --- | --- | --- | --- | --- |
|  | M/G | G/M | M/G | G/M | M/G | G/M |
| infralimbic cortex | 0.144±0.026 | 0.101±0.023 | 0.163±0.031 | 0.088±0.014 | 0.129±0.017 | 0.098±0.023 |
| ventral orbital cortex | 0.105±0.016 | 0.062±0.022 | 0.161±0.045 | 0.044±0.013 | 0.134±0.028 | 0.054±0.011 |
| NAc | 0.0925±0.014 | 0.058±0.027 | 0.138±0.055 | 0.033±0.004 | 0.11±0.027 | 0.046±0.009 |
| piriform  cortex | 0.095±0.0165 | 0.03±0.007 | 0.104±0.048 | 0.027±0.007 | 0.133±0.027 | 0.039±0.006 |
| septum | 0.164±0.033 | 0.039±0.009 | 0.135±0.032 | 0.024±0.008 | 0.115±0.02 | 0.037±0.006 |
| striatum | 0.1±0.012 | 0.097±0.036 | 0.1±0.02 | 0.045±0.011 | 0.1±0.021 | 0.083±0.024 |
| thalamus | 0.044±0.018 | 0.007±0.006 | 0.02±0.005 | 0.002±0.001 | 0.028±0.007 | 0.003±0.001 |
| SpVN | 0.008±0.003 | 0.001±0.0003 | 0.008±0.004 | 0.0002±0.0002 | 0.01±0.002 | 0.001±0.001 |
| NTSc | 0.12±0.023 | 0.013±0.005 | 0.068±0.015 | 0.006±0.001 | 0.142±0.035 | 0.012±0.004 |
