# Supplementary material for: Longitudinal Tracking of Astrocyte Reactivity During the Development of Chronic Orofacial Neuropathic Pain Using [ 18F]‐SMBT‐1 Positron‐Emission Tomography
Source: Glia. 2026 Jun 18;74(8):e70182. doi: 10.1002/glia.70182 (PMC13278361; doi:10.1002/glia.70182)
Supplement: Supplementary file 10 — Table S7: Body weight change throughout experimental timeline, physiological changes, and anesthetic monitoring within the positron emission tomography scanner at each experimental time ION‐CCI (n = 11), Sham (n = 6), and Naïve (n = 8) groups. [file GLIA-74-0-s006.docx]

**Supplementary table 7.** Body weight change throughout experimental timeline, physiological changes, and anaesthetic monitoring within the positron emission tomography scanner at each experimental time ION-CCI (n=11), Sham (n=6), and Naïve (n=8) groups.

|  | **group** | | | **single Factor ANOVA** | |
| --- | --- | --- | --- | --- | --- |
|  | **ION-CCI (mean±SEM)** | **Sham (mean±SEM)** | **Naïve (mean±SEM)** | **F-stat** | **p-value** |
| **body weight (grams)** | | | | | |
| day -7 | 230.91±4.88 | 250.83±12.43 | 239.63±4.33 | 1.78 | 0.19 |
| day +2 | 260.36±4.43 | 288.33±12.50 | 281.50±3.46 | 4.68 | 0.02 |
| day +7 | 286.27±8.60 | 297.17±5.66 | 301.75±4.86 | 1.13 | 0.34 |
| day +14 | 305.00±5.04 | 339.33±11.40 | 326.38±5.09 | 5.90 | 0.009 |
| day +28 | 355.54±5.67 | 379.00±13.58 | 370.13±7.83 | 1.79 | 0.19 |
| **respiratory change (BPM)** | | | | | |
| day -7 | -1.88±1.50 | -3.50±2.89 | -2.00±2.44 | 0.11 | 0.89 |
| day +2 | -0.55±2.94 | 0.67±3.02 | -1.63±2.72 | 0.11 | 0.89 |
| day +7 | 4.82±2.63 | 1.67±2.94 | -0.63±2.66 | 0.97 | 0.40 |
| day +14 | 4.82±3.10 | 3.00±3.1 | -2.13±2.75 | 0.91 | 0.42 |
| day +28 | 4.55±2.68 | 3.00±2.69 | 5.25±3.28 | 0.63 | 0.54 |
| **temperature change (°C)** | | | | | |
| day -7 | -0.50±0.18 | -0.18±0.08 | -0.68±0.26 | 1.02 | 0.38 |
| day +2 | -0.28±0.28 | -0.40±0.20 | 0.04±0.20 | 0.62 | 0.54 |
| day +7 | -0.45±0.13 | -0.17±0.10 | -0.26±0.11 | 1.21 | 0.32 |
| day +14 | -0.18±0.09 | -0.07±0.17 | -0.15±0.13 | 0.18 | 0.84 |
| day +28 | -0.24±0.22 | -0.25±0.19 | -0.09±0.09 | 1.35 | 0.28 |
| **average anaesthetic dose (%)** | | | | | |
| day -7 | 1.94±0.08 | 1.96±0.14 | 1.96±0.08 | 0.01 | 0.99 |
| day +2 | 2.10±0.10 | 2.05±0.17 | 2.11±0.12 | 0.05 | 0.96 |
| day +7 | 1.93±0.09 | 1.96±0.19 | 2.14±0.10 | 0.80 | 0.46 |
| day +14 | 1.96±0.09 | 2.07±.017 | 2.22±0.12 | 1.24 | 0.31 |
| day +28 | 1.88±0.09 | 1.88±0.15 | 2.03±0.12 | 0.51 | 0.61 |
| **number of anaesthetic dose changes** | | | | | |
| day -7 | 3.38±0.37 | 3.17±0.44 | 3.33±0.40 | 0.05 | 0.95 |
| day +2 | 3.18±0.40 | 3.33±0.65 | 2.63±0.25 | 0.60 | 0.56 |
| day +7 | 3.18±0.38 | 2.83±0.44 | 3.50±0.35 | 0.52 | 0.60 |
| day +14 | 3.64±0.41 | 2.67±0.19 | 2.88±0.33 | 1.76 | 0.20 |
| day +28 | 3.55±0.43 | 3.00±0.58 | 2.50±0.25 | 1.45 | 0.25 |
