# Supplementary material for: Longitudinal Tracking of Astrocyte Reactivity During the Development of Chronic Orofacial Neuropathic Pain Using [ 18F]‐SMBT‐1 Positron‐Emission Tomography
Source: Glia. 2026 Jun 18;74(8):e70182. doi: 10.1002/glia.70182 (PMC13278361; doi:10.1002/glia.70182)
Supplement: Supplementary file 11 — Table S8: [18F]‐SMBT‐1 time activity curve values within candidate reference regions: Cerebellum, left and right thalamus, and infralimbic cortices. Each value represents the mean ± SEM standardized SMBT‐1 uptake value (SUV) within these sites in each experimental cohort: Naïve, Sham or ION‐CCI rats, averaged across all five experimental time points. [file GLIA-74-0-s004.docx]

|  | **cerebellum** | | | **left thalamus** | | | **right thalamus** | | | **infralimbic cortex** | | |
| --- | --- | --- | --- | --- | --- | --- | --- | --- | --- | --- | --- | --- |
| **time (mins)** | **ION-CCI** | **Sham** | **Naïve** | **ION-CCI** | **Sham** | **Naïve** | **ION-CCI** | **Sham** | **Naïve** | **ION-CCI** | **Sham** | **Naïve** |
| 1 | 3.84±0.16 | 3.84±0.32 | 3.84±0.12 | 3.52±0.12 | 3.56±0.25 | 3.95±0.13 | 3.72±0.20 | 3.85±0.31 | 4.18±0.14 | 3.54±0.16 | 3.33±0.34 | 3.82±0.13 |
| 2 | 3.18±0.07 | 3.20±0.16 | 3.81±0.07 | 3.55±0.11 | 3.54±0.17 | 3.85±0.06 | 3.57±0.12 | 3.73±0.21 | 3.41±0.05 | 3.42±0.14 | 3.49±0.27 | 3.62±0.10 |
| 3 | 2.60±0.05 | 2.55±0.12 | 3.20±0.06 | 3.08±0.08 | 3.03±0.14 | 3.27±0.08 | 3.17±0.10 | 3.18±0.14 | 2.76±0.05 | 2.88±0.05 | 2.78±0.19 | 3.10±0.09 |
| 4 | 2.22±0.02 | 2.21±0.10 | 2.90±0.06 | 2.71±0.07 | 2.71±0.12 | 2.89±0.04 | 2.80±0.07 | 2.73±0.13 | 2.45±0.04 | 2.52±0.07 | 2.40±0.13 | 2.63±0.03 |
| 5 | 2.00±0.04 | 1.92±0.08 | 2.62±0.06 | 2.51±0.05 | 2.32±0.09 | 2.56±0.05 | 2.42±0.03 | 2.41±0.11 | 2.18±0.04 | 2.16±0.04 | 2.16±0.12 | 2.37±0.05 |
| 7 | 1.75±0.03 | 1.68±0.04 | 2.20±0.02 | 2.04±0.02 | 2.03±0.08 | 2.20±0.05 | 2.08±0.05 | 1.99±0.05 | 1.96±0.03 | 1.85±0.03 | 1.81±0.06 | 2.01±0.06 |
| 9 | 1.51±0.02 | 1.44±0.04 | 1.86±0.02 | 1.72±0.02 | 1.64±0.04 | 1.85±0.03 | 1.72±0.03 | 1.67±0.06 | 1.63±0.01 | 1.59±0.03 | 1.53±0.07 | 1.75±0.05 |
| 11 | 1.34±0.02 | 1.26±0.04 | 1.68±0.04 | 1.53±0.02 | 1.45±0.07 | 1.64±0.04 | 1.48±0.03 | 1.45±0.05 | 1.47±0.02 | 1.35±0.02 | 1.32±0.03 | 1.49±0.03 |
| 13 | 1.19±0.02 | 1.13±0.03 | 1.49±0.02 | 1.34±0.02 | 1.26±0.04 | 1.44±0.03 | 1.34±0.01 | 1.25±0.04 | 1.31±0.02 | 1.21±0.02 | 1.17±0.03 | 1.35±0.03 |
| 15 | 1.07±0.02 | 1.02±0.04 | 1.34±0.03 | 1.22±0.02 | 1.15±0.04 | 1.30±0.05 | 1.18±0.02 | 1.13±0.02 | 1.22±0.02 | 1.08±0.02 | 1.07±0.04 | 1.20±0.02 |
| 20 | 0.93±0.01 | 0.89±0.03 | 1.14±0.02 | 1.03±0.01 | 0.97±0.05 | 1.11±0.01 | 1.01±0.02 | 0.94±0.03 | 1.06±0.01 | 0.94±0.02 | 0.89±0.03 | 1.06±0.02 |
| 25 | 0.78±0.01 | 0.72±0.03 | 0.95±0.03 | 0.85±0.01 | 0.80±0.03 | 0.91±0.02 | 0.83±0.02 | 0.78±0.04 | 0.88±0.01 | 0.78±0.02 | 0.74±0.03 | 0.87±0.02 |
| 30 | 0.66±0.01 | 0.62±0.02 | 0.79±0.02 | 0.70±0.01 | 0.67±0.03 | 0.78±0.02 | 0.70±0.01 | 0.65±0.03 | 0.75±0.02 | 0.65±0.01 | 0.61±0.02 | 0.75±0.03 |
| 40 | 0.52±0.01 | 0.50±0.02 | 0.63±0.02 | 0.57±0.01 | 0.52±0.02 | 0.63±0.02 | 0.57±0.01 | 0.53±0.03 | 0.60±0.01 | 0.52±0.01 | 0.52±0.02 | 0.59±0.01 |
| 50 | 0.40±0.01 | 0.38±0.01 | 0.47±0.01 | 0.43±0.01 | 0.42±0.02 | 0.47±0.02 | 0.43±0.01 | 0.40±0.02 | 0.46±0.01 | 0.40±0.01 | 0.37±0.02 | 0.44±0.01 |
| 60 | 0.31±0.01 | 0.29±0.01 | 0.37±0.02 | 0.33±0.01 | 0.31±0.01 | 0.37±0.01 | 0.33±0.01 | 0.32±0.01 | 0.36±0.01 | 0.30±0.01 | 0.30±0.01 | 0.35±0.02 |
| ***decay Constant at each experimental time point (*λ)** | | | | | | | | | | | | |
| **day -7** | 0.044 | 0.044 | 0.043 | 0.041 | 0.044 | 0.043 | 0.043 | 0.043 | 0.043 | 0.044 | 0.043 | 0.044 |
| **day +2** | 0.039 | 0.044 | 0.041 | 0.037 | 0.043 | 0.039 | 0.037 | 0.043 | 0.041 | 0.039 | 0.041 | 0.040 |
| **day +7** | 0.041 | 0.045 | 0.042 | 0.039 | 0.039 | 0.039 | 0.040 | 0.044 | 0.040 | 0.040 | 0.043 | 0.041 |
| **day +14** | 0.044 | 0.036 | 0.041 | 0.041 | 0.040 | 0.039 | 0.041 | 0.041 | 0.039 | 0.042 | 0.041 | 0.042 |
| **day +28** | 0.042 | 0.039 | 0.038 | 0.040 | 0.038 | 0.035 | 0.041 | 0.038 | 0.035 | 0.040 | 0.038 | 0.034 |

**Supplementary table 8.** [^18^F]-SMBT-1 time activity curve values within candidate reference regions: Cerebellum, left and right thalamus, and infralimbic cortices. Each value represents the mean±SEM standardized SMBT-1 uptake value (SUV) within these sites in each experimental cohort: Naïve, Sham or ION-CCI rats, averaged across all five experimental time points.
