# Supplementary material for: Longitudinal Tracking of Astrocyte Reactivity During the Development of Chronic Orofacial Neuropathic Pain Using [ 18F]‐SMBT‐1 Positron‐Emission Tomography
Source: Glia. 2026 Jun 18;74(8):e70182. doi: 10.1002/glia.70182 (PMC13278361; doi:10.1002/glia.70182)
Supplement: Supplementary file 12 — Table S9: Average initial uptake and decay of [18F]‐SMBT‐1 whole brain SUV within ION‐CCI, Naïve, and Sham cohorts. Single factor ANOVA was conducted to assess between group variance, with coefficient of variation testing performed to assess within‐group variance of initial tracer uptake. [file GLIA-74-0-s010.docx]

**Supplementary table 9.** Average initial uptake and decay of [^18^F]-SMBT-1 whole brain SUV within ION-CCI, Naïve, and Sham cohorts. Single factor ANOVA was conducted to assess between group variance, with coefficient of variation testing performed to assess within-group variance of initial tracer uptake.

|  | Mean±SEM | | | p-value |
| --- | --- | --- | --- | --- |
|  | ION-CCI (n=11) | Naïve (n=8) | Sham (n=6) | Single Factor ANOVA |
| Dynamic Frame 1 | 3.32±0.13 | 3.45±0.24 | 2.93±0.22 | 0.21 |
| Delta Dynamic Frame 1-2 | 0.32±0.08 | 0.28±0.05 | 0.21±0.11 | 0.64 |
|  |  |  |  |  |
| Coefficient of Variation | 13.6% | 19.1% | 19.2% |  |
